# Growth and development of the third permanent molar in *Paranthropus robustus* from Swartkrans, South Africa

**DOI:** 10.1038/s41598-020-76032-2

**Published:** 2020-11-04

**Authors:** Christopher Dean, Clément Zanolli, Adeline Le Cabec, Mirriam Tawane, Jan Garrevoet, Arnaud Mazurier, Roberto Macchiarelli

**Affiliations:** 1 Department of Earth Sciences, Natural History Museum, London, UK; 2 Department of Cell and Developmental Biology, University College London, London, UK; 3 Univ. Bordeaux, CNRS, MCC, PACEA, UMR 5199, 33600 Pessac, France; 4 Department of Maxillofacial and Oral Surgery, Sefako Makgatho Health Sciences University, Ga-Rankuwa, Pretoria, South Africa; 5 Department of Human Evolution, Max Planck Institute for Evolutionary Anthropology, Leipzig, Germany; 6 Ditsong National Museum of Natural History, Pretoria, South Africa; 7 Deutsches Elektronen-Synchrotron DESY, Hamburg, Germany; 8 IC2MP, UMR 7285 CNRS, Université de Poitiers, Poitiers, France; 9 UMR 7194 CNRS, Muséum National D’Histoire Naturelle, Musée de L’Homme, Paris, France; 10 Unité de Formation Géosciences, Université de Poitiers, Poitiers, France

**Keywords:** Biophysics, Evolution, Anatomy

## Abstract

Third permanent molars (M3s) are the last tooth to form but have not been used to estimate age at dental maturation in early fossil hominins because direct histological evidence for the timing of their growth has been lacking. We investigated an isolated maxillary M3 (SK 835) from the 1.5 to 1.8-million-year-old (Mya) site of Swartkrans, South Africa, attributed to *Paranthropus robustus*. Tissue proportions of this specimen were assessed using 3D X-ray micro-tomography. Thin ground sections were used to image daily growth increments in enamel and dentine. Transmitted light microscopy and synchrotron X-ray fluorescence imaging revealed fluctuations in Ca concentration that coincide with daily growth increments. We used regional daily secretion rates and Sr marker-lines to reconstruct tooth growth along the enamel/dentine and then cementum/dentine boundaries. Cumulative growth curves for increasing enamel thickness and tooth height and age-of-attainment estimates for fractional stages of tooth formation differed from those in modern humans. These now provide additional means for assessing late maturation in early hominins. M3 formation took ≥ 7 years in SK 835 and completion of the roots would have occurred between 11 and 14 years of age. Estimated age at dental maturation in this fossil hominin compares well with what is known for living great apes.

## Introduction

The most easily observable and widely employed measure of dental maturation in modern humans, dental eruption, is in fact one of the least reliable^1,2^. Paradoxically, however, molar eruption has been the measure of maturation most often used in comparative studies of humans with great apes and with fossil hominins^3–8^. Mineralised tooth tissues (enamel and dentine), on the other hand, contain daily increments of growth that are often well-preserved, even in fossil teeth^9,10^. These have been used to calibrate the eruption times of first permanent molars (M1s) in fossil hominins and this has proved to be a reasonably successful method for assessing the time and patterning of dento-skeletal maturation in a broad comparative context^6,11–16^, if perhaps a less successful proxy for other life history variables^17–20^. Nonetheless, despite the fact that greater numbers of late juvenile and sub-adult fossil hominins of unknown chronological age are now associated with skeletal material approaching maturity^21–28^, assessing the end of the tooth maturation process remains problematic, as too few data exist to reconstruct the timing of late third permanent molar (M3) development. Indeed, to date, the age at completion of the “wisdom tooth”, i.e., the measure of late dental maturity, has never been directly reconstructed, but only inferred in australopiths (*Australopithecus* and *Paranthropus*) and early *Homo*^29,30^.

The initial mineralisation of the M3 tooth in both great apes and early fossil hominins occurs at a chronologically younger age than in modern humans. In *Pan* and *Gorilla,* the age range reported, albeit for relatively few individuals (Supplementary Text S1), spans almost 2.5 years, from 2.9 to 5.3 years^31–37^, but for a large number of modern humans the range is much greater, at least 7 years, from 5 to 12 years^38–41^. Radiographically, in great apes, either a large empty crypt in the jaw bone, or a minute mineralising M3 cusp tip within a crypt, appears close to the time of true M3 initiation. This happens as the premolars and second permanent molars (M2s) complete enamel formation (between the radiographically-defined fractional stages of tooth Crown ¾ complete and Root initiation) and as the first permanent molars (M1s) are close to root completion (between radiographic stages Root ¾ formed and Apex ½ closed) (Supplementary Table S1, Supplementary Figs. S1–S3).

Just one fossil hominin, the Early Pleistocene StW 151 from Sterkfontein, South Africa, variably attributed to early *Homo*^42^ or *A. africanus*^43^, or even considered as taxonomically indeterminate^16^, shows evidence of a large empty M3 crypt, and is aged 4.62–4.70 years^16,42^. Unfortunately, the age at death of another fossil specimen with an empty M3 crypt, the Early Pleistocene early *Homo* KNM-ER 820 from Koobi Fora, Kenya^44,45^, is yet to be determined.

Clearly, there is insufficient evidence to determine a mean age for M3 initiation in early hominins. However, the comparative information increasingly available about the timing and sequence of these events in great apes and fossil specimens, notably for the stages of dental development both before and after M3 initiation^16,37^, strongly suggests M3 initiation occurred in australopiths and early *Homo* sometime between 4 and 7 years of age. Early hominin specimens aged ~ 4 years or less at death, such as the SK 63 *P. robustus* specimen from Swartkrans, or the *A. africanus* Taung child, both from South Africa, show no evidence of M3 initiation^16,46–49^. Even with a possible 3-year age range for M3 initiation, knowing the time taken for M3 crown and root formation in an early hominin would allow us to address a number of issues, including the likely age range at dental maturation.

Here we present the first histological data for the timing of M3 development in an early hominin. SK 835 is a maxillary left M3 attributed to the hominin taxon *P. robustus* and was recovered from the Early Pleistocene cave site of Swartkrans^50^. Following high-resolution virtual scanning and 3D imaging performed by X-ray micro-tomography (µXCT; Supplementary Fig. S4), we made thin ground sections through the crown and roots that captured the whole of tooth growth (Supplementary Texts S2–S4, Supplementary Figs. S5–S7).

We used a combination of transmitted light microscopy (TLM) and synchrotron X-ray fluorescence (SXRF) to image the daily incremental growth markings in enamel and dentine. SXRF of this specimen revealed that apparently random bands of Sr enrichment were laid down in the crown and roots of this tooth during development. These, together with other prominent accentuated markings in enamel and dentine visible in TLM, were used to align the cusps and roots of SK 835 that formed at the same time. In this way we cross-matched daily incremental growth markings from different locations within the tooth and reconstructed the growth processes involved in enamel and dentine formation. One aim of this study was to shed light on the physical nature of the circadian growth increments preserved in the enamel and dentine.

There is now good evidence that circadian clocks operate in ameloblasts and odontoblasts^51–54^ and that they regulate amelogenin secretion and other ameloblast activities^55^ as enamel prism formation cycles through alternating fast and slow phases^56–61^. How exactly this circadian rhythm manifests as a fine dark cross striation across the narrow slowly formed constriction of a prism body is however less clear^55,56^. Cross striations are more porous^62^ and show a compositional contrast in back-scattered electrons-based (BSE) microscopic images that has been attributed to increased carbonate content^56^, or perhaps to an alternating dominance of matrix over mineral deposition during the day^55^. It is also likely that abrupt changes in enamel crystallite orientation and/or continuity contribute to the appearance of cross striations in TLM and in polarised light microscopy (PLM)^63,64^. Raman microscopy has previously shown that the peak intensity of OH, Ca^2+^ and PO^4-^ occurs at the prism varicosity and that the intensity of all three ions co-vary in a cyclic manner^65^.

Daily incremental markings in enamel and dentine enable the rates and timing of crown and root formation to be retrieved from fossil teeth. A second aim of this study was thus to quantify the changing daily rates of enamel and dentine secretion throughout the entire growth of the SK 835 early hominin M3. A further aim was to put a time scale to the fractional stages of tooth formation commonly used in comparative radiographic and other imaging studies of modern humans, great apes and fossil hominins^28,37,38,66,67^. Since tooth growth is a continuous and seamless process, we also reconstructed growth curves describing cuspal enamel secretion and the increase in tooth length along the once developmental boundaries between tissues, the enamel–dentine junction (EDJ) and the cementum–dentine junction (CDJ), and compared these with similar data for living hominids and fossil hominins.

## Results

### The nature of daily incremental markings

**Figure 1:**
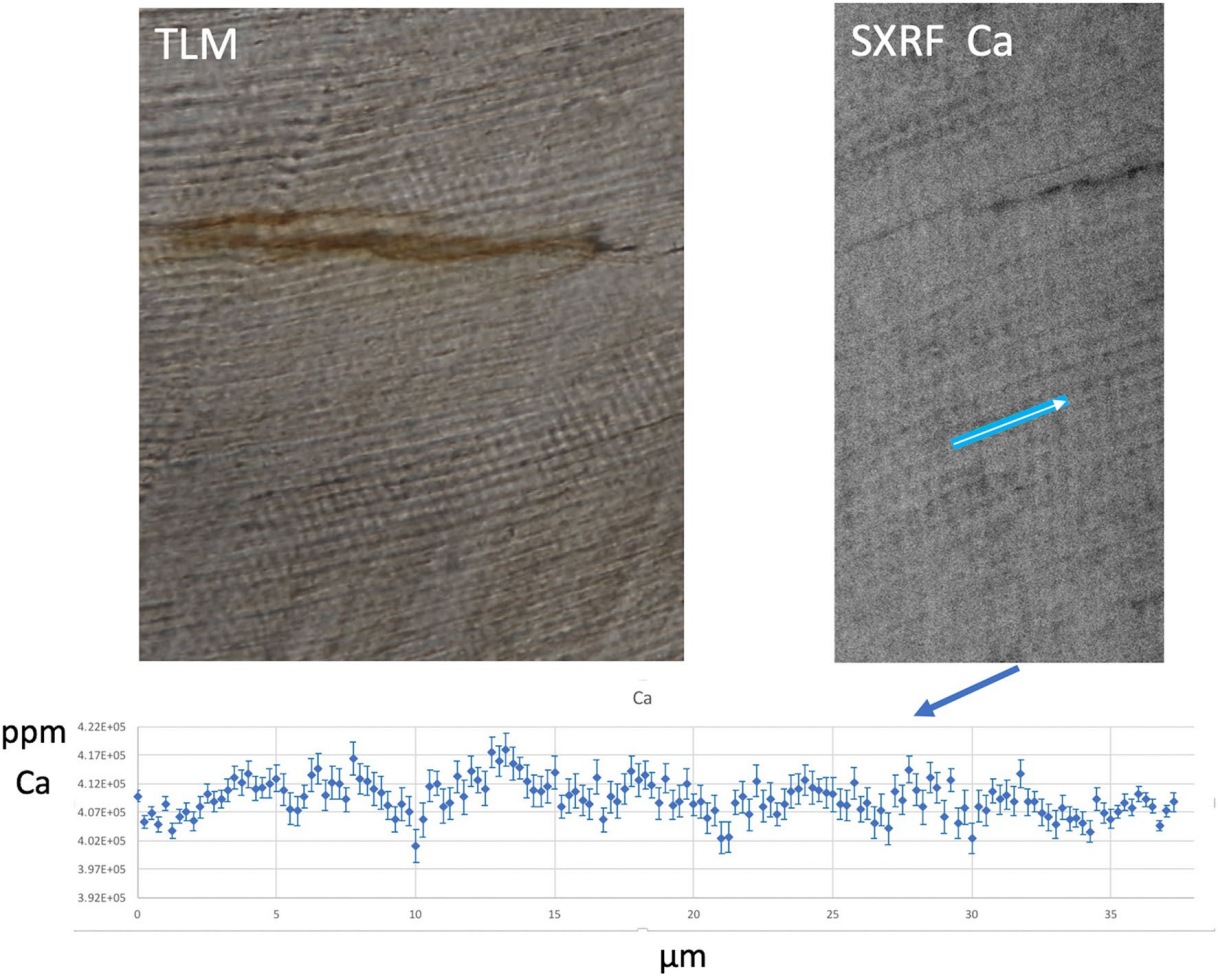
Transmitted light micrograph (TLM) of cervical enamel in the SK 835 protocone showing a region with clear daily incremental markings (left image). A SXRF (*synchrotron X-ray fluorescence*) image in the same general region showing periodic fluctuations in Ca concentration. The plot across the blue transect is ~ 35 µm long and shows ~ 7 peaks and troughs (from left to right) in Ca concentration that fluctuate between 402,000 and 417,000 ppm. Error bars associated with each datapoint represent SD. The blue line represents the scan transect. (200 × 100 µm^2^, 17.0 keV, 0.25 µm, 10 ms, scan time 1 h).

**Figure 2:**
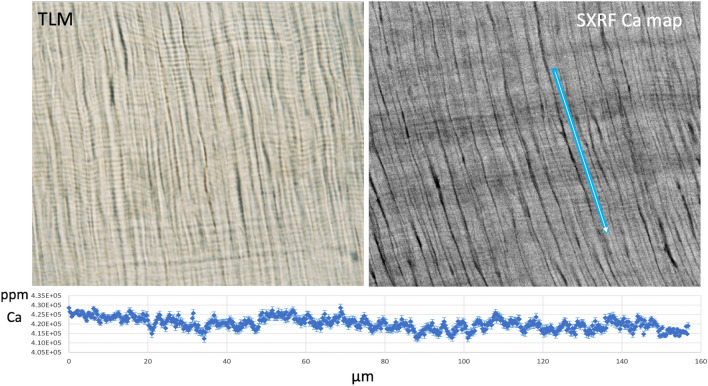
Transmitted light micrograph (TLM) of daily dentine increments in the apical root dentine of the palatal root of SK 835 (left image). A SXRF image of Ca concentration of the dentine in the same general region. The blue line is ~ 160 µm long and shows the transect represented in the plot of Ca concentration (from top to bottom) against distance (µm). Error bars associated with each datapoint represent SD. Across more than 30 incremental markings, Ca concentration fluctuated between 410,000 and 430,000 ppm but broader zones of lower Ca concentration spanned daily increments in some regions. (300 × 300 µm^2^, 17.0 keV, 0.25 µm, 10 ms, scan time 4.5 h).

Two regions of SK 835 that in TLM showed clear daily enamel cross striations and daily dentine increments, were scanned with SXRF (at 0.25 µm resolution). In both tissues, the alternating light and dark increments visible in TLM corresponded most strongly with fluctuations in Ca concentration (Figs. 1 and 2). This suggests the brighter increments seen in TLM are relatively mineral dense and the darker increments less dense, which is consistent with previous BSE-based findings^56,62^. Broader irregular accentuated markings were also associated with more diffuse shifts in Ca concentration that spanned the underlying daily rhythms (Figs. 1 and 2). The regular daily changes in Ca concentration do not, however, occur abruptly at cross striations in the SXRF scans but more closely resemble the circadian fluctuations reported^68^ for plasma ionized Ca^2+^. Interestingly in this SXRF study, unlike previous studies^65^, there was no discernible variation in phosphorus concentration (a major component of hydroxyapatite) where the circadian rhythm has been reported to peak twice in a day^68^.

### Rates of enamel formation

**Figure 3:**
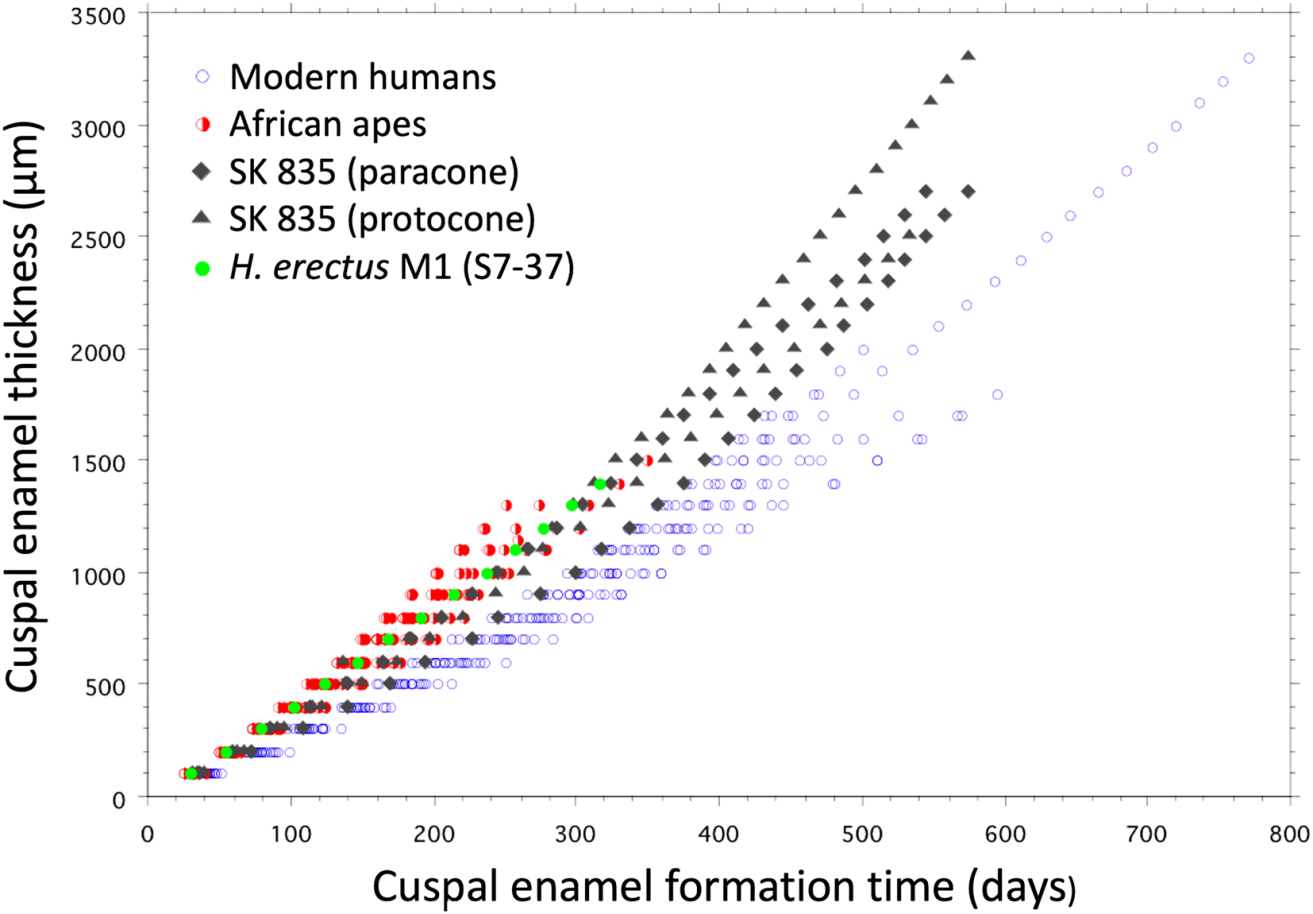
Plot showing increase of enamel thickness against enamel formation time (days). For each 100-µm thickness of cuspal enamel, the number of daily increments were counted along prism tracks and summed. In SK 835 the trajectories between the EDJ and the enamel surface are shown as grey filled diamonds (paracone) and triangles (protocone). The lowest of these is the buccal paracone trajectory that overlaps with data for modern humans of all three molar types pooled (n = 20, open blue circles). The trajectories for SK 835 are similar to a sample of African apes of all three molar types pooled (n = 12 *Pan*, n = 8 *Gorilla*, red half-filled circles) and a single M1 specimen of *H. erectus* (S7-37) (filled green circles). Thick cuspal enamel in SK 835 continues to form over a longer period of time than in African apes and early *H. erectus*. Modern human molars follow a slower trajectory. This results from slower inner rates of enamel secretion continuing at a slow rate for a longer time. Cusps may grow thick or thin along either trajectory. One human molar (M3) is exceptionally thick ~ 3,300 µm and took more than 2 years to form cuspal enamel. Daily cross striation spacings in the same human M3 are also profiled in Supplementary Fig. S10.

Four prism tracks were defined in the first-formed cuspal enamel of the SK 835 crown (Supplementary Fig. S7). Cumulative counts of daily increments were made at 100 µm intervals through the whole thickness of enamel, as well as measurements of cross striation spacings within ten successively formed 60-day zones of cuspal enamel formation (Supplementary Text S3, Supplementary Figs. S7–S11). Some surface enamel was lost through wear, but 575–580 daily increments are still preserved in the tallest protocone cusp of this *P. robustus* M3. Formation rates ranged between 3.2 µm/day, close to the EDJ, and rose to 8.28 µm/day before reducing again to ~ 7.3 µm/day close to the enamel surface. Formation rates in modern human molars rarely exceed 6.5 µm/day in outer cuspal enamel, but can also take 600–700 days to form thick molar cuspal enamel, as was likely the case in SK 835 (Supplementary Figs. S8–S11). The gradient of enamel formation through the cusps of this upper M3 are greater than measured in a sample of modern human molars (Fig. 3), but similar to that in some living great apes and other early hominin molars^69–71^.

By combining the four trajectories of the defined prism tracks, the average time (Y) taken to form a given thickness of occlusal enamel (X) in the SK 835 *P. robustus* tooth can be predicted from the equation (Eq. 1) given here (see Supplementary Text S3 for 95% confidence intervals):

1 $${\text{Y }} = { 12}.{651 } + \, .{\text{284X }} - { 3}.{\text{459e}}^{{ - {5}}} {\text{X}}^{{2}} \left( {{\text{R}}^{{2}} = \, .{979}} \right).$$

### Growth in tooth length

**Figure 4:**
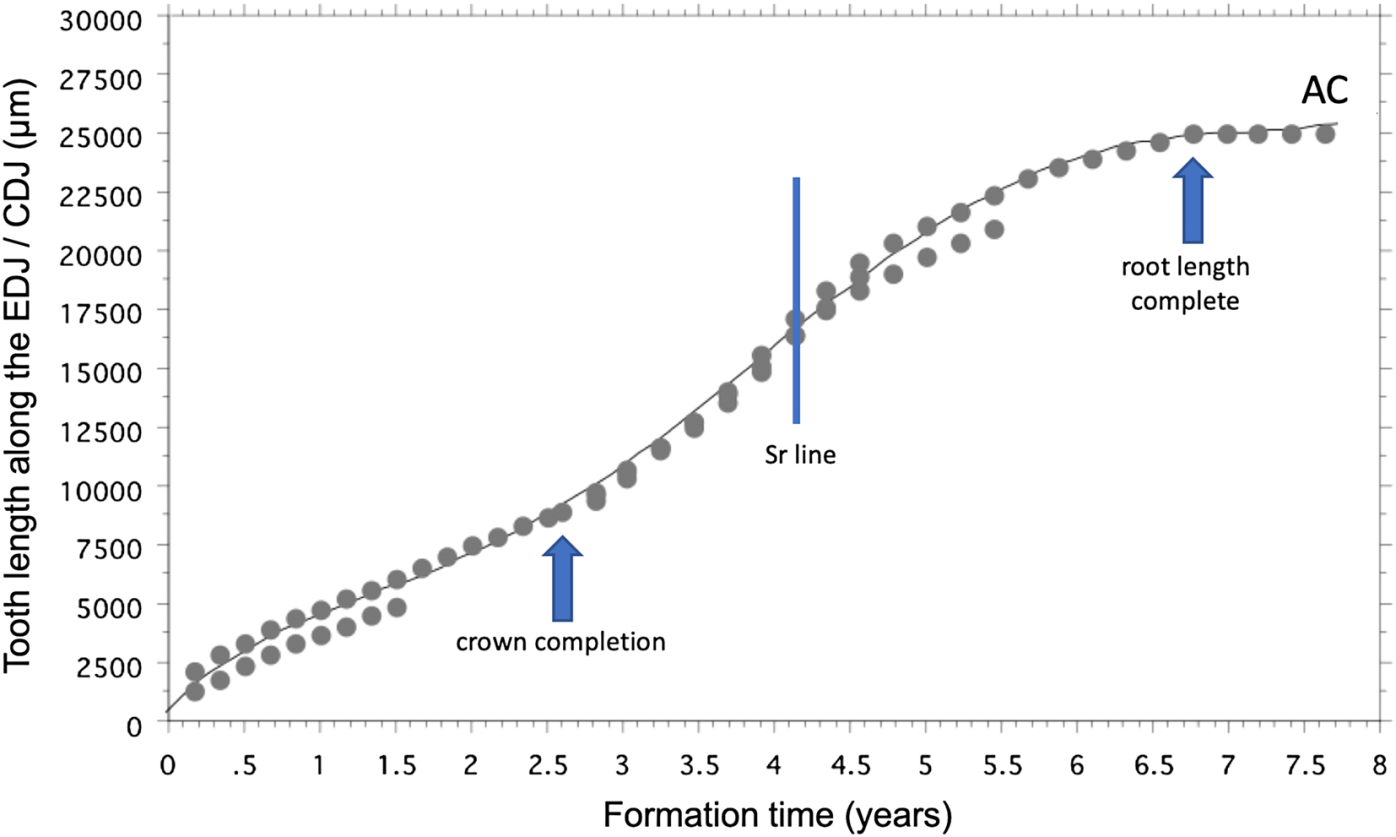
Combined plot of increase in tooth length in both cusps along the EDJ and then up to crown completion in the protocone (arrow at 2.59 years) and continuing beyond this along the CDJ in the damaged palatal root and then onwards to root length complete in the distobuccal root (arrow at 6.75 years). The roots are cross-matched at 4.12 years (Sr line). The plot is extended here to apex closure (AC) at 7.75 years. Each of the component crown and root plots are combined as a single best-fit cumulative growth curve. The plot shows increasing distance along the EDJ/CDJ (µm) against formation time (years) and is expressed here by a 5th order polynomial that allows formation time (Y) to be predicted from a known value of EDJ/CDJ length (X). Although apex closure is extended to 7.75 years here, it may have taken less time. Predictions, using the following formula, of tooth formation time for each successive 1 mm of growth in length along the EDJ/CDJ are also given in Supplementary Table S2: (Eq. 2) Y = .018 + 8.875e^−5^X + 4.414e^−8^X^2^ − 3.009e^−12^X^3^ + 4.581e^−17^X^4^ + 6.494e^−22^X^5^; R^2^ = .993.

**Figure 5:**
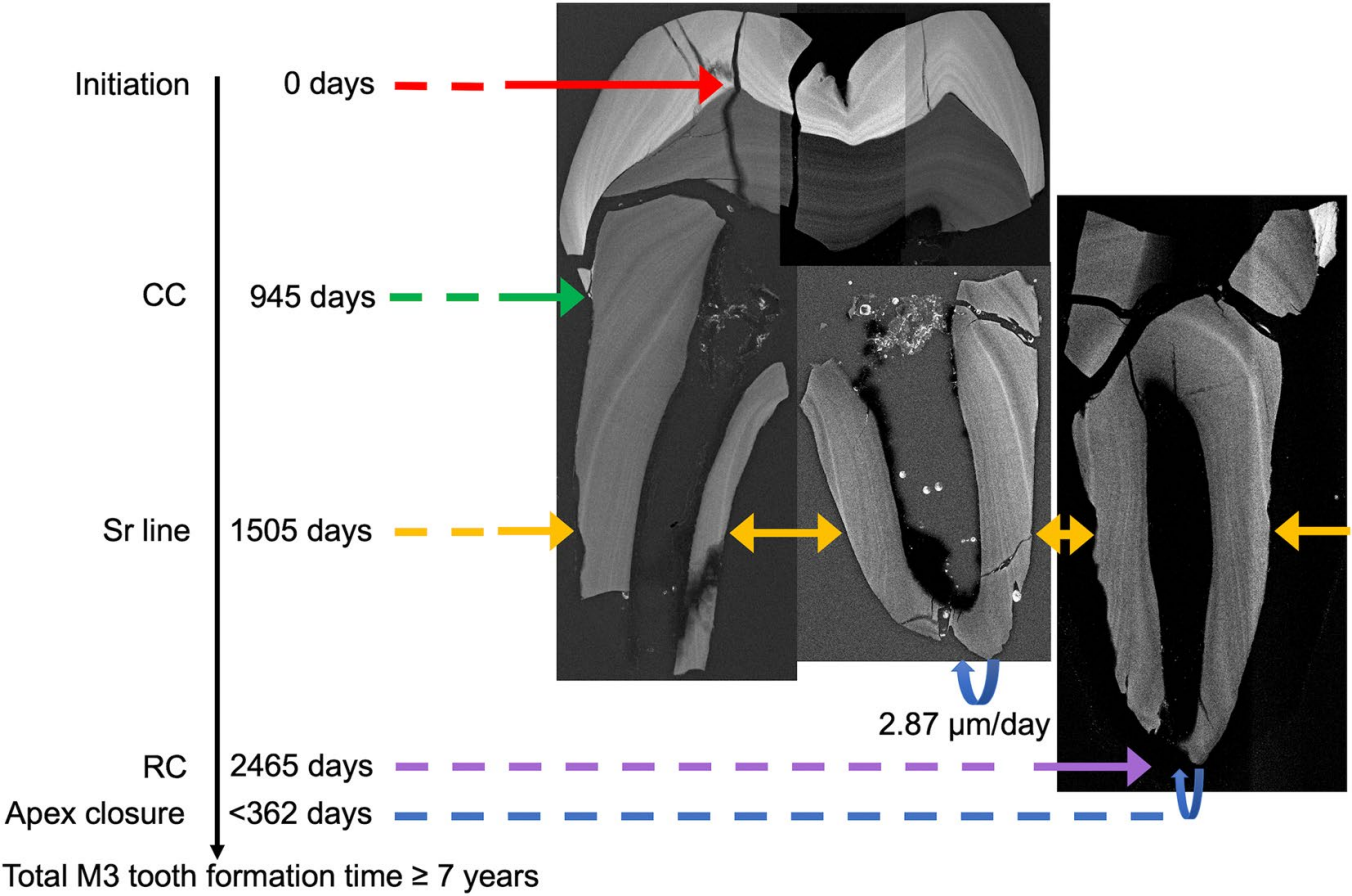
Mosaic of five SXRF montaged images showing Sr concentration (intensity) of three ground sections of SK 835. These are cross-matched to a prominent Sr line in the three separate roots (yellow arrows) that formed at 4.1 years (1505 days) into M3 formation. Tooth formation (initiation) is denoted at zero days (red arrow) in the protocone. The formation times for crown completion (945 days, green arrow) and the stage of tooth root length complete at 2465 days, (RC, purple arrow) are derived from the histological data for daily increments of growth in the crown and root and rates of root extension. Daily rates of dentine formation in the mesiobuccal root apex (2.87 µm/day) were used to estimate the time taken to complete closure of the distobuccal root apex (< 362 days, blue dotted line). Total tooth formation time is estimated to have been ≥ 7 years.

Teeth grow in length as cohorts of secretory cells differentiate in continuity, first along the boundaries between the EDJ in the crown, then beyond this in the root between the CDJ. The rate of differentiation (the extension rate), can be reconstructed from the daily formation rate and the slope of incremental or accentuated lines that represent the former position of the ameloblast or odontoblast cell sheet^72–76^. To calculate the time and rate of extension in SK 835 between initial mineralisation of the tooth and completion of its root length, we used the average times taken to form an initial 200 µm thickness of enamel (61 days) or dentine (80 days), and the orientation of incremental markings to the EDJ or CDJ, respectively (Supplementary Texts S3–S4, Supplementary Figs. S12–S14). This was possible between protocone initiation to the end of its damaged palatal root. It was also possible in the preserved part of the paracone and separately in the detached distobuccal root (Fig. 5, Supplementary Text S4, Supplementary Fig. S7). Since each of these preserved components contain an intense SXRF Sr marker-line (Fig. 5), that formed at 4.1 years into tooth formation, they could be cross-matched in time and the cumulative tooth length plotted against formation time (Fig. 4).

For any given length (X) along the EDJ/CDJ, formation time (Y) in this *P. robustus* maxillary M3 can be predicted from the equation (Eq. 2) given here:

2 $${\text{Y }} = \, .0{18 } + { 8}.{\text{875e}}^{{ - {5}}} {\text{X }} + { 4}.{\text{414e}}^{{ - {8}}} {\text{X}}^{{2}} - { 3}.00{\text{9e}}^{{ - {12}}} {\text{X}}^{{3}} + { 4}.{\text{581e}}^{{ - {17}}} {\text{X}}^{{4}} + { 6}.{\text{494e}}^{{ - {22}}} {\text{X}}^{{5}} \left( {{\text{R}}^{{2}} = \, .{993}} \right).$$

### Fractional stages of tooth growth

For modern humans and many non-human primates, defined fractions of tooth formation in cross-sectional studies have proved a more practical way of compiling chronological growth standards than longitudinal growth curves for each tooth type^1,2,37,66,67^. However, they remain just snapshots of a continuous and seamless growth process.

**Figure 6:**
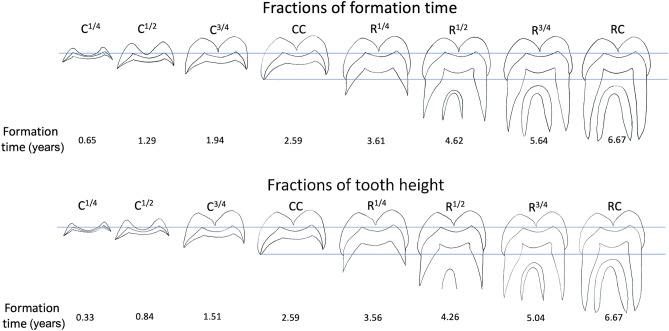
Diagrammatic representations of a *P. robustus* M3, based on SK 835, showing successive ¼ fraction stages of time between crown initiation (Ci) and root completion (RC) as well as ¼ fraction stages of crown height and root height. Either stages of time fractions or of height fractions can be matched to any developing hominin M3, which ever fits best, and to the formation time estimated for SK 835 (given here in years). Adding a 3-year range (from 4 to 7 years) for M3 initiation times to the age given for any fractional stage provides a range of chronological ages for dental development that, although wide, may bracket the undoubted variation there would be in M3 growth and development generally. Supplementary Table S4 lists 21 Plio-Pleistocene hominins with incomplete M3s. Note that crown completion (CC) and root completion (RC) are identical in both formation stage sequences.

Rates of tooth growth in early hominins differ from those compiled for modern humans and so the age standards for the fractional stages defined for modern humans do not compare^77–79^. To reconstruct the most commonly used fractional stages of increasing tooth length (quarters of crown height and quarters of root length) for SK 835, we used the pattern of Sr banding and the accentuated markings in enamel and dentine together with the time scale derived from daily incremental markings. Figure 5 summarises the key formation times of each component in this fossil M3. In addition, we reconstructed new stages of tooth formation that represent the amount of tooth formed in quarters of crown and root formation time (Fig. 6; Supplementary Fig. S15). The last stage of root formation represented in Fig. 6 is root length completed (RC), that was attained in SK 835 at 6.67 years into M3 formation. The equation for cumulative increase in EDJ/CDJ length also allows M3 formation time in SK 835 to be predicted for any given length (Supplementary Table S2).

Using the evidence for root apex closure in SK 835 derived from its shorter mesiobuccal root to reconstruct final completion in the longer and last formed root apexes, which are damaged, is problematic, but a reasonable estimate is that this may have taken a year, or so, to close (Supplementary Text S4). A safe conclusion is that the whole of M3 formation in SK 835, from the dentine horn in the crown to root apex closure, would have taken no less than 7 years, and possibly longer.

## Discussion

The estimate of ≥ 7 years for the time taken to grow an M3 in a representative of *P. robustus*, based on the data collected from the upper third molar specimen SK 835 from the 1.5–1.8 Mya site of Swartkrans, South Africa, represents the first direct evidence of this kind for any early hominin. Given a likely 4–7-year age range for M3 initiation, it now becomes possible to say that completion of the permanent dentition and root apex closure in SK 835 would have occurred sometime between 11 and 14 years of age. This estimate appears to compare well with what is known for dental maturation in living great apes^19,32,34,37,80–82^.

With respect to somatic growth, it has been suggested *P. robustus* showed a gorilla-like pattern of extended male growth (bimaturism)^83^. By assuming that, in principle, among samples of early hominins larger specimens are more likely males and the smaller ones females^84^, one could argue that SK 835 more likely represents a female individual. Indeed, crown size in SK 835, especially for the mesiodistal diameter (M-D: 13.9 mm) but, to a lesser extent, also for the buccolingual diameter (B-L: 16.6 mm)^50^, is lower than the average values reported for the *P. robustus* upper M3s sample from Swartkrans (*n* = 16; M-D: 14.9 mm, range 12.7–17.2 mm; B-L: 17.0 mm, range 15.9–18.2 mm)^85^, and also lower, but only for the M-D diameter, than measured in the assemblage of *P. robustus* M3s from the South African sites of Swartkrans, Kromdraai and Drimolen (*n* = 25; M-D: 14.5 mm, range 12.1–17.2 mm; B-L: 16.5 mm, range 14.2–18.2 mm)^86^. Nonetheless, given the extent of tooth crown size variation, and especially of the “wisdom tooth”, this remains a hazardous exercise for all early hominins, including *P. robustus*^87^.

Despite the large range of daily enamel secretion rates in M3 cuspal enamel reported in this study (3.28–8.28 µm/day), the average cuspal rate in SK 835 (overall protocone average 5.56 µm/day, SD = 1.63; overall paracone average 4.89 µm/day, SD = 1.24) compares well with the data for KB 5223, another *Paranthropus* specimen^43^ from the Early Pleistocene site of Kromdraai B (5.05 µm/day) but which has also previously been attributed to early *Homo*^88^. In general, these cuspal rates also compare well with the average estimates provided for *P. robustus* (5.7 µm/day)^89^ and with those previously determined for other fossil hominins such as StW 151 (5.53 µm/day) and early *Homo* (6.06 µm/day)^16^. Previously published data for inner cuspal enamel formation rates in *P. robustus* (4.07–4.25 µm/day) have, however, tended to be higher^89–91^ than measured in SK 835 (3.28 µm/day). In fact, the inner rates of occlusal enamel formation reported here for SK 835 align better with rates previously reported and summarised by Lacruz et al.^71^ for *Pan troglodytes* (3.62 µm/day), *A. afarensis* (3.31 µm/day), *P. aethiopicus* (3.5 µm/day), *P. boisei* (2.94 µm/day), *H. rudolfensis* (3.01 µm/day), *H. habilis* (3.68 µm/day), and *H. erectus* (3.05 µm/day). When comparing such data sets, much depends on how close to the EDJ cross striation spacing measurements have been possible and on the different definitions of inner cuspal enamel. These new results for SK 835 do however suggest it may be premature to use summary differences in rates of enamel formation rather than gradients through time to support taxonomic differences^55,92^.

Ramirez Rozzi^79^ has calculated that secretory ameloblasts in thick enamelled molars attributed to *P. boisei* continue to form cuspal enamel for a greater proportion of the total crown formation time (53.8%, range in *n* = 14 M3s, 43–73%) than, for example, in thinner enamelled gorillas (15–23%, *n* = 8 molars). In SK 835, cuspal enamel in the protocone continued to form for 580/945 days, or 61% of the total crown formation time. Enamel thickness seems likely to be an adaptation to extend the chewing-lifespan of a tooth in the face of an abrasive diet^93^, but average enamel thickness (AET) is also a major component of absolute crown strength (ACS) that confers resistance to fractures associated with mechanically resistant foods^94^. The weaving (or decussation) of enamel prisms as they pass from the EDJ to the enamel surface is also a crack-stopping adaptation^95^, but strong decussation, especially close to the EDJ, is one factor that slows the rate at which cuspal enamel thickness accumulates. Prisms in *P. boisei* and *P. robustus* enamel run straighter for longer and form at a faster rate through cuspal enamel and this creates a different pattern of more tightly spaced Hunter-Schreger bands than in early *Homo* and modern humans^85,96^. This narrow banding pattern may be one manifestation of a trade-off to both form thick enamel resistant to a lifetime’s abrasion in as short a time as possible, but which also confers maximal fracture-resistant properties with minimal decussation. However, despite *P. robustus* and modern humans both exhibiting relatively thick-enamelled molars (i.e., similar relative enamel thickness [RET] values; Supplementary Text S5, Supplementary Table S3, Supplementary Fig. S16), this condition is achieved in different ways. Molar cuspal enamel close to the EDJ in modern humans forms more slowly for a longer period of time than in *P. robustus* (Fig. 3) and other australopiths^70^. Cuspal enamel formation rates in *P. robustus* (SK 835) immediately start to increase from the EDJ and reach rates in the outer enamel that exceed those in modern humans (Supplementary Figs. S9–S10). Together with differences in crown shape, this relatively thick but faster-formed cuspal enamel in *P. robustus,* confers a higher crown resistance to fractures in *P. robustus* M3s than in modern humans (as assessed by the ACS index; Supplementary Table S3).

**Figure 7:**
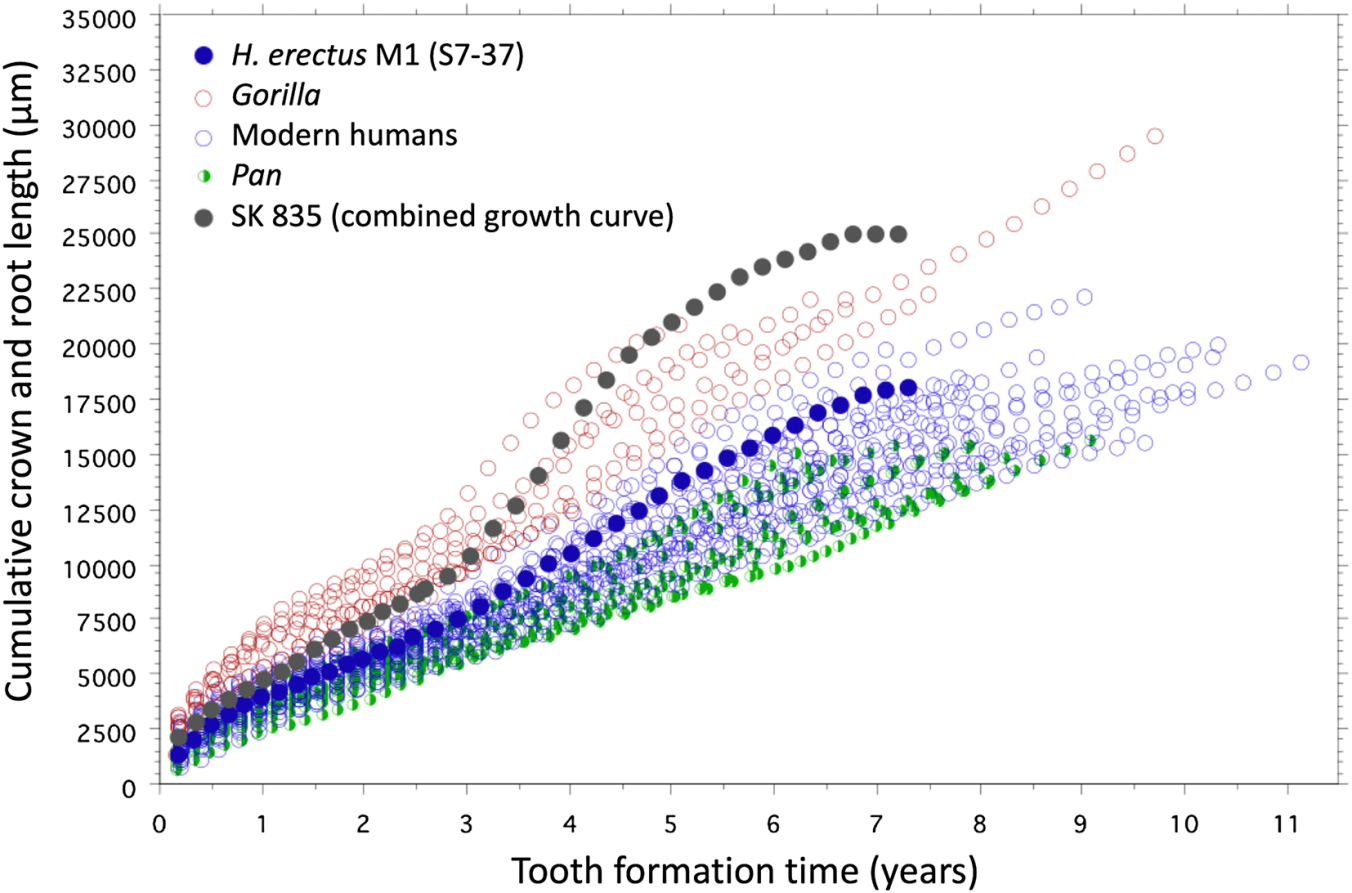
Increase in cumulative tooth length against tooth formation time. The plot shows increasing distance along the EDJ/CDJ (µm) against formation time (years) for a sample of modern human molars (M2, n = 8; M3, n = 8; blue open circles), *Pan* M3s (n = 10; green half-filled circles) and *Gorilla* (M1, n = 2; M2, n = 2; M3, n = 2; red open circles), data from^9,75^. Superimposed onto these extant taxa are SK 835 (black filled circles) and an M1, S7-37 from Java, attributed to *H. erectus*, (dark blue filled circles), data from^70,75^. Increase in crown height along the EDJ in SK 835 overlaps with some modern humans and some *Gorilla* specimens. However, root growth in SK 835, that begins at 2.59 years, stands out as faster than all but a few *Gorilla* M2s and M3s. Despite having considerably longer roots than, for example the *H. erectus* M1 root, completion in these two contrasting fossil molars occurs at approximately the same time in ~ 7 years from tooth initiation since rates of root extension are faster in SK 835.

Crown formation time in SK 835 (2.59 years; Figs. 5 and 6; Supplementary Text S3) compares well with previous estimates of 2.12–2.59 years of molar crown formation times in *P. boisei* from Koobi Fora, Kenya^77^, and with those of 2.7 years estimated for *n* = 14 Plio-Pleistocene hominin M3s from the Omo Valley, Ethiopia^79^. Both the crown formation time of SK 835 and the total tooth formation time (≥ 7 years) also compare well with the only other fossil hominin molar for which the whole of tooth formation has been reconstructed histologically: the S7-37 upper M1 attributed to *H. erectus* from the Early Pleistocene site of Sangiran, Java^97^. In this latter specimen, crown formation took 2.6 years and total tooth formation time 7.3 years^70,98,99^. Figure 7 shows that the difference in growth between SK 835 and the Indonesian fossil hominin molar lies largely in the fast root extension rates that set the *P. robustus* tooth apart from extant humans, *Pan* and *Gorilla* as well as this single representative of *H. erectus*. Considered here in a comparative context, SK 835, with absolutely longer tooth roots, shows faster rates of root extension, and as far as can be judged from a limited sample size, most closely resembles molar tooth root growth in gorillas.

It must of course be acknowledged that these new data for M3 formation times, derived from the analysis of the *P. robustus* specimen SK 835 from Swartkrans, are for a single tooth and that the age range for M3 initiation among early hominins remains uncertain. However, they provide another line of evidence for assessing maturation during late development in fossil hominins and, importantly, one that is independent of standards established for great apes and/or modern humans^1,2,37,66,67^. Specifically, if the 3-year range of initiation times and stages of M3 development in SK 835 were to encompass those in other fossil hominins, they would bracket the still debated chronological age at death in TM 1517, the holotype of *P. robustus* where M3 root formation is at stage R¾^28^, to between 9.04 and 12.04 years. Extending these kind of age estimates to other *P. robustus* specimens with incomplete M3s seems reasonable in the absence of any other direct evidence, although age estimates for other early fossil hominins remain speculative.

Supplementary Table S4 lists some key specimens with incompletely formed M3s. For one thing it demonstrates how many early fossil hominins are known with incompletely formed M3s but for which no age at death estimates, however broad, have been possible using the M3. Based on the present results obtained for SK 835, a range of age at death estimates, all be it a wide range, is now possible when virtual histology^15,16^ or other approaches are either impossible or impractical, or pending. Notably, the MH1 Early Pleistocene *A. sediba* from Malapa, South Africa, and another individual with a well-preserved partial skeleton attributed to *H. erectus* from West Turkana, Kenya (KNM-WT 15000), both have a complete or near-complete M3 crown^14,24,30,100^. Based on the findings of this study, these early hominins would both fall within the age range 6.59–9.59 years. Moreover, a further *H. erectus* specimen with associated postcranial elements from the Early Pleistocene site of Dmanisi, Georgia (D2700/D2735), with approximately half of the M3 root formed, would fall within the age range 8.26–11.26 years^23,101^. Based on the evidence from SK 835, it is possible to state that completion of the permanent dentition and root apex closure in early hominins generally would have been between 11 and 14 years of age.

The data presented here also allow us to re-examine previous studies of M3s in early hominins that have focused on australopith paleobiology and behaviour. Especially relevant are the contextual issues relating to australopith habitat, mobility, and growth^102,103^. Previous research using strontium isotope analysis suggested that *P. robustus* had a chimpanzee-like residence pattern implying male philopatry and female exogamy, i.e., with the adolescent females emigrating from their natal groups^102^. More recently, Sillen and Balter^103^ measured the ratio ^87^Sr/^86^Sr in eight M3s from Swartkrans, all attributed to *P. robustus* but of undiagnosed sex. Given that their laser ablation scan-tracks closely follow the cuspal/occlusal trajectories defined in this study, and based on our results for cuspal enamel formation times in SK 835, it can be now estimated that these laser ablation tracks are likely to span 500–600 days of enamel formed during the first half of M3 crown formation. Assuming a 3-year range for M3 initiation in *P. robustus*, this suggests that only enamel formed between ~ 5.5 and 8.5 years of age was sampled in all eight individuals examined by Sillen and Balter^103^. It is significant that all individuals showed ^87^Sr/^86^Sr signatures consistent with having lived and died in their immediate locality at these ages, with no evidence of having moved in from elsewhere^103^. However, it is clear from this study that 5 or 6 years of M3 development still remained and, as Sillen and Balter^103^ pointed out, this more than ever now leaves open questions about seasonal and/or permanent spatial displacement of *P. robustus* individuals across the differing landscapes during the later stages of M3 development. The presence of such clear Sr banding in the roots of SK 835 (Fig. 5) suggests the prospect of answering such questions is promising.

The data for M3 development presented in this study fill a gap in our knowledge about late dental development in early hominins. They provide a new and independent line of evidence for assessing the age at death of sub-adult specimens. They also demonstrate the potential of combining high-resolution elemental analysis with more traditional histological analysis of fossil tooth tissues.

## Methods

### X-ray micro-tomography (µXCT)

The microtomographic acquisition of SK 835 was performed in 2015 at the PLATINA platform of the IC2MP set at the (University of Poitiers, France), with the EasyTom XL Duo laboratory device (RX-solutions, France). A sealed microfocus X-ray source (L12161-07, Hamamatsu Photonics, Japan) was used coupled to a flat panel detector (PaxScan 2520DX, Varian, USA). The specimen was scanned at a spatial resolution of 25 µm according to the following acquisition parameters: 70 kV (tube voltage); 350 µA (tube current); 1184 projections; 9.3 frames per second; averaging of 15 frames per projection; filtration of the beam by a 1.2 mm aluminium filter; a source-to-detector distance and a source-to-object distance of 368 mm and 72.5 mm, respectively. We applied a procedure using random shifts of the detector to avoid the presence of ring artefacts on the reconstructed images. We also recorded 32 reference images at the end of the microtomographic scan to correct for possible beam drift. The data were reconstructed in 16 bits TIF images with the XAct software (RX-solutions). For the reconstruction, we applied a filtered back projection algorithm based on the Feldkamp method for cone beam geometry associated with a Tukey filter and beam hardening artefacts were corrected through linearization with a polynomial function.

### Image processing, virtual reconstruction and enamel thickness assessment

A semiautomatic threshold-based segmentation of SK 835 was conducted using Avizo v.8.0 (FEI Visualization Sciences Group, Hillsboro), following an adaptation of the half-maximum height method^104^. The segmented tooth fragments were then refitted together using landmarks on corresponding aspects to reconstruct the original tooth and a constrained smoothing was applied to generate its 3D surface (Supplementary Fig. S4). Linear and surface measurements of enamel, crown dentine and pulp were taken on the virtual buccolingual section passing through the mesial dentine horns. Two bidimensional indices assessing enamel thickness were then computed based on these variables: the relative enamel thickness index (2D RET) and the absolute crown strength (ACS)^94^. Data for SK 835 were compared with microtomographic-based records of M3s representing *P. robustus* (original data), modern humans (original data), *Pan* (^105^ and collections of the Institut Català de Paleontologia Miquel Crusafont), *Gorilla*^105^, and *Pongo* (Ref.^105^, collections of the Institut Català de Paleontologia Miquel Crusafont, Museo di Storia Naturale di Trieste, Musée Zoologique de Strasbourg).

### Histological section preparation

SK 835 consists of three separate crown fragments and three separate root fragments (Supplementary Text S2, Supplementary Figs. S4–S7). These were all first cleaned, scanned and imaged using µXCT (see above), and then either partially reassembled for sectioning or, in the case of two roots, sectioned separately. The dentine horns within the cusps were located with µXCT images to help define best planes of histological section. Histological methods are also provided in Dean et al.^106,107^. Crown and root fragments were sectioned longitudinally with a low speed diamond saw (*Buehler IsoMet*). The cut block face that contained the best plane of section was lightly lapped through a series of silicon carbide abrasive papers (*Buehler CarbiMet,* P800-P1200) and then taken to a mirror finish using 1 µm aluminium oxide powder (*Buehler, MicroPolish*) and deionised water on a felt polishing pad (*Buehler ChemoMet*). Polishing and cutting residue was cleared from the block in an ultrasonic bath. After 24 h in a silica gel desiccator, the polished block face was fixed under pressure for 48 h to a 1 mm thick glass slide using a low viscosity slow-curing dual component epoxy resin designed for glass (*Huntsman Araldite 2020*). A second cut was then made parallel with the block face/glass slide leaving ~ 300 µm tooth tissue attached to the slide. This was lapped plane-parallel to ~ 100 µm in a hand-held extrudable steel slide holder (*Buehler*) first on fine wet abrasive papers (P2500) and then for final polishing on a soft polishing cloth (*Buehler MicroCloth*) with a 1 µm diamond suspension (*MetaDi Poly 1* *µm*) to ensure no surface contamination (e.g., with alumina powder) that might affect SXRF scans. While SXRF scans were performed without a coverslip, sections were cleared in xylene and mounted with *DPX* (Distyrene, Phthalate plasticiser in xylene) for transmitted light microscopy.

### Synchrotron X-ray fluorescence (SXRF) imaging

Experiments were performed on the P06 Beamline^108,109^, Petra III, at DESY (Deutsches Elektronen-Synchrotron, Hamburg, Germany, a member of the Helmholtz Association HGF). The storage ring was operated in 40-bunch mode using top-up filling mode with a current of 100 mA ± 0.5 mA. The primary X-ray beam was monochromatised to 17.0 keV using a double crystal Si111 monochromator and focused using a Kirkpatrick-Baez (KB) mirror system (*JTEC, Japan*) to 500 × 500 nm^2^. For this experiment, the set-up comprised a Maia 384C detector system^110^, ideally used in “backscatter” geometry to maximise the solid angle during analysis of thin polished samples (~ 100 µm-thick in this study) and allowing for large area SXRF imaging with a sub-µm resolution using millisecond dwell times^111^. Further details on the set-up are provided in Dean et al.^106,107^. Elements of primary interest were Ca and Sr. Spectral analysis, deconvolution and initial image analysis of the fluorescence data were performed using GeoPIXE 7.4f.^112^. The X-ray yield calculations were performed assuming a hydroxyapatite matrix (Ca_10_(PO_4_)_6_(OH)_2_) with density 3.1 g/cm^3^ close to enamel^113^ and final sample thickness of 80–100 µm. Glass slides were included in the overall sample model as appropriate. Concentrations were determined using a conversion factor (photon counts to equivalent charge) through measurement of a standard Ni foil with areal density 50.0 µg/cm^2^ (*Micromatter Technologies Inc. Canada*). Elemental distribution maps were normalised to the incoming X-ray flux. SXRF concentrations are reported as parts-per-million (ppm or µm/g).

Overview scans of the mesiobuccal and distobuccal roots of SK 835 were made at 10 µm, 10 ms, and each took 5.0 h. Scans of the central cuspal/occlusal enamel, the protocone and palatal root together, and the paracone, were also performed at 10 µm, 10 ms, and took between 1.3 and 5.5 h. Two regions of interest were then scanned to image daily enamel cross striations (at 0.25 µm 10 ms and 1 h) and daily dentine increments (at 0.25 µm 10 ms and 4.5 h). Using GeoPIXE 7.4f., two sets of elemental profiles for Ca were made along transects defined on the SXRF scans of enamel and dentine daily increments (Figs. 1 and 2).

### Histological methods

Data for daily rates of enamel and dentine formation were collected with TLM (Supplementary Texts S3–S4). Groups of ameloblasts secret prisms (long bundles of hydroxyapatite crystallites) that extend from the EDJ to the enamel surface. The change in spacing (or repeat interval) of daily enamel cross striations along prisms was measured and counted through enamel in SK 835 to provide rates and times of cuspal enamel formation. Repeated measurements across groups of daily increments in enamel and dentine were made at multiple locations within a 200-µm zone of the EDJ or CDJ and an average value was used as the rate of formation for this given thickness of tissue. Previous methods of calculating total enamel formation times^58,59,72,114^ were adapted to estimate the rate at which ameloblasts and odontoblasts differentiated, or extended, across the whole length of the crown and/or root^73–75,115^. At the start of crown or root formation, a line was projected from the EDJ or CDJ (point ‘a’ in Supplementary Fig. S14) along the direction of enamel or dentine apposition to a point 200 µm deep (point ‘c’ in Supplementary Fig. S14). Accentuated incremental markings representing the slope of the original forming cell sheet were identified at point ‘c’ and traced back to, but further along, the EDJ or CDJ in the direction of crown and root formation (point ‘b’ in Supplementary Fig. S14). The rate of increase in crown or root length (defined as the extension rate^72^) equals the distance between points ‘a’ and ‘b’ divided by the time taken to form the 200-µm thickness of tissue between ‘a’ and ‘c’ (Supplementary Fig. S14). This procedure was repeated consecutively to the end of tooth formation and the cumulated data plotted as a growth curve.

## Supplementary information

Supplementary Information.

## Data Availability

Raw images and derived data supporting the findings of this study are available from the corresponding author (C.D.) on request.
