# Supplementary material for: Growth and development of the third permanent molar in Paranthropus robustus from Swartkrans, South Africa
Source: Sci Rep. 2020 Nov 4;10:19053. doi: 10.1038/s41598-020-76032-2 (PMC7642444; doi:10.1038/s41598-020-76032-2)
Supplement: Supplementary file 1 — Supplementary Information. [file 41598_2020_76032_MOESM1_ESM.pdf]

SUPPLEMENTARY INFORMATION

Growth and development of the third permanent molar in *Paranthropus robustus* from Swartkrans, South Africa

Christopher Dean[1,2*], Clément Zanolli[3,4], Adeline Le Cabec[3,5], Mirriam Tawane[6], Jan Garrevoet[7], Arnaud Mazurier[8] & Roberto Macchiarelli[9,10]

[1]Department of Earth Sciences, Natural History Museum, London, UK. [2]Department of Cell and Developmental Biology, University College London, London, UK. [3]Univ. Bordeaux, CNRS, MCC, PACEA, UMR 5199, F-33600 Pessac, France. [4]Department of Maxillofacial and Oral Surgery, Sefako Makgatho Health Sciences University, Ga-Rankuwa, Pretoria, South Africa; [5]Department of Human Evolution, Max Planck Institute for Evolutionary Anthropology, Leipzig, Germany. [6]Ditsong National Museum of Natural History, Pretoria, South Africa. [7]Deutsches Elektronen-Synchrotron DESY, Hamburg, Germany. [8]IC2MP, UMR 7285 CNRS, Université de Poitiers, Poitiers, France. [9]UMR 7194 CNRS, Muséum national d'Histoire naturelle, Musée de l'Homme, Paris, France. [10]Unité de Formation Géosciences, Université de Poitiers, Poitiers, France. *email: ucgacrd@ucl.ac.uk

Supplementary Information includes:

- Supplementary Texts S1-S5
- Supplementary Tables S1-S4
- Supplementary Figures S1-S17
- Supplementary References

Supplementary Text S1: M3 initiation times

Radiographic stages. M3 development is the most variable of all tooth types. The time of initiation of M3 mineralisation can be determined either radiographically or from histology. Radiographically, a large empty crypt appears first, followed by the mineralising cusp tips. True initiation lies somewhere between these two radiographic stages but can never be determined precisely from radiographs. Radiographically, mean age of crypt appearance in modern humans ranges from 7.33 to 9.26 years[1,2] and mean age of M3 initiation between 7.92 to 9.69 years, but the range of ages is huge and may be from 5 to 12 years.

For the purposes of this study, 14 great apes, 11 of which were wild-shot museum specimens, with either a large empty lower M3 crypt or a crypt containing one or more mineralising cusp tips, were scored for radiographic formation stages of the premolars and first and second permanent molars in the same jaw quadrant (all radiographs are published in [3–5]). Where an empty M3 crypt was visible, or mineralising M3 cusps were already present, premolars and second permanent molars were between radiographic stages Crown complete and Root ¼ formed. The M1 was consistently between radiographic stages Root ¾ formed and Apex ½ closed (Supplementary Table S1, Supplementary Figs S1-S3).

In great apes of known age, the observed range of M3 initiation is tighter than in modern humans, likely in part because this occurs earlier in the growth period. For 5 captive chimpanzees of known age, the range was 2.99 to 4.61 years (mean: 3.5 years, SD: 0.64)[6], and for a further 12 chimpanzees the mean was 3.64 years (SD: 0.33)[7].

M3 cusp initiation was well underway in a free-living mountain gorilla at 5.31 years[8]. Reid and Guatelli-Steinberg[9] have noted that histological examination usually reveals earlier evidence of tooth initiation and estimated more advanced M3 initiation in 3 modern humans (range: 5.46-6.6 years). In comparing their data with early fossil hominins, they noted that it seemed likely early hominins more closely resembled modern humans in their range of initiation times than living great apes, "perhaps suggesting a grade shift in initiation times, at least for posterior teeth, early in human evolution"[9].

Not much direct information is available from the early hominin fossil record. Only a small incipient M3 crypt is visible in a ~4-year-old early hominin (SK 63) from Swartkrans[10] and a large empty crypt is present in a 4.62-4.70-year-old early hominin (StW 151) from Sterkfontein[11–13]. The same is true in a slightly chronologically older early *Homo* mandible (KNM-ER 820) from Koobi Fora, Kenya[14]. No ages for M3 initiation in early hominins have ever been published. Many early hominins, however, do exist with M3s at later stages of development. On the basis of the evidence reviewed here, it seems clear there is insufficient evidence to determine a mean M3 initiation age for early hominins, or to improve on the broad estimate of Beynon and Dean[15] of ~5 years of age. However, a near-identical pattern, or sequence of premolar, M1 and M2 formation stages is observed in great apes and the few early hominins close to the stage of M3 initiation. Importantly, alongside this, new information is now available about the timing of events in premolar, M1 and M2 development, both earlier and later than M3 initiation in great apes and early hominins[8,12,13] and suggests M3 initiation occurred sometime between 4 and 7 years of age in early hominins.

Supplementary Text S2: Specimen description

The crown of SK 835, a maxillary left M3, consists of three separate fragments that can be fitted together with reasonable accuracy (Supplementary Fig. S5). Two mesial fragments comprise the paracone and protocone, and a third fragment comprises the distal metacone and hypocone. Each of the three roots are also separate from each other and the crown, but can be joined together at the base of the crown, although there is considerable damage around the cervix and root trunk. The palatal root consists of a coronal segment that extends

up within the enamel cap, with a small portion of the last-formed cervical enamel attached to it. This is the only true continuity between the crown and root. The palatal root is, however, incomplete and fractured obliquely across its apical third. The mesiobuccal root is incomplete at the buccal cervix and cannot be joined to the crown, but it does preserve the root apex on both its mesial and lingual surfaces. The distobuccal root is made up of three small cervical fragments, two palatal and one buccal, previously glued together. The buccal fragment includes a small portion of cervical enamel marking the crown-root junction. This root is almost complete to the apex buccally, but is damaged lingually (Supplementary Figs S5-S7).

The two mesial crown fragments (paracone and protocone), along with the palatal root, were located together in the best fit possible using cyanoacrylate cement. The dentine horns within both mesial cusps were identified on µXCT scans and the points above them marked on the enamel surface overlying these (Supplementary Fig. S4). A plane of section was identified that passed through both paracone and protocone dentine horns and through the long axis of the palatal root (Supplementary Fig. S5). A decision was made to section the mesiobuccal and distobuccal roots separately. Buccolingual longitudinal planes of section through the mesiobuccal and distobuccal roots were defined with the intention of maximising the root width, root canal and root length captured within each section (Supplementary Figs S6, S7). The enamel and dentine surfaces along the planes of section were coated with cyanoacrylate cement and allowed to dry for 48 hours to prevent any brittle tissue flaking off during the cutting and polishing process. This is also easily dissolved off post-sectioning enabling easy reconstruction of the cleaned blocks whereas embedding the whole tooth in, for example, methyl methacrylate, risks greater damage when de-embedding is required.

Supplementary Text S3: Enamel histology

Cuspal enamel formation time and rate. Digital photo-montages were constructed of the SK 835 enamel cap. Four prism paths were identified in cuspal enamel where good daily prism cross striations could be tracked from the EDJ to the enamel surface. These are indicated in sequence from left (palatal) to right (buccal) in Supplementary Figure S7 by the four red arrows 'a'-'d'. These were: (a) palatal to the protocone dentine horn, 1200 µm along the EDJ avoiding poorly preserved enamel; (b) directly over the protocone dentine horn, alongside a crack in this position; (c) directly over the paracone dentine horn; and (d) immediately buccal to the axial paracone dentine horn.

Decussation in the enamel was minimal in outer enamel but still apparent, especially in inner enamel. Each straight-line prism path was divided into 100-µm segments or zones and direct counts of daily enamel cross striations that weave from side to side within and between zones were made and cumulated. The total cumulative counts along each prism path between zero days at the EDJ and the last-formed occlusal enamel at the surface were respectively 580, 533, 555 and 575 days, with the greatest counts on the palatal aspect of the protocone (580 days) and buccal of the paracone (575 days). However, only the first prism track (580 days) emerges at an unworn enamel surface. For all four prism path trajectories combined, a second order polynomial regression plot describes the cumulative days of enamel formation (Supplementary Fig. S8). Note that in Supplementary Figure S8 the distance from the EDJ is plotted on the Y axis, but in the equations that follow, Y corresponds to the formation time in days.

For all four trajectories combined, the time (Y) taken to form a given thickness of occlusal enamel (X) equals:

$$Y = 12.651 + .284X - 3.459e^{-5}X^2 \ (R^2 = .979). \qquad \text{(Eq. 1)}$$

Lower 95% confidence interval:

$$Y = -.197 + .265X - 4.063e^{-5}X^2.$$

Upper 95% confidence interval:

$$Y = 25.498 + .303X - 2.856e^{-5}X^2.$$

When the slower palatal protocone trajectory is excluded, the following second order polynomial regression plot describes the average cumulative days of enamel formation across the remaining three trajectories:

$$Y = 18.029 + .276X - 2.866e^{-5}X^2 \ (R^2 = .987)$$

To provide a better sequential profile of variation in daily enamel formation rates through the cuspal enamel, 60-day zones were then defined between the EDJ and the enamel surface along each of the four prism paths. Within each 60-day zone, groups of 6 cross striations were marked that define 5 days enamel growth. The average linear daily rate of enamel formation was calculated by dividing the distance along a prism from the first to the sixth cross striations by 5. This was repeated as many times as possible within each 60-day zone, where a minimum 8 times and a maximum 23 times were possible. While it would have been desirable to divide the cuspal enamel into smaller 30-day (monthly) zones, in some places too few measurements were possible to generate meaningful box plots.

The profile of changing enamel rates through these 60-day zones is shown in Supplementary Fig. S9; that of rate changes in a modern human M1, M2 and M3 from the same individual, but divided into 30-day zones, appears for comparison in Supplementary Fig. S10 and illustrates the typical M1-M3 declining gradient in modern humans but with similar rates at the EDJ. In SK 835, the protocone and paracone cusp tips are both slightly worn, so, apart from the most palatally positioned prism path in the protocone, no true outer enamel remains. Average rates close to the EDJ were 3.28 µm/d and, in the protocone, rose to 8.28 µm/d (SD: 0.406, range: 7.5-9.0 µm/d) in outer enamel before reducing again to ~7.3 ± 0.5 µm/d in the last 120 days of enamel formation at the surface, which became aprismatic here.

When summarised as inner, middle and outer regions of equally thick cuspal enamel, the overall mean values for all cross striation spacings within the palatal aspect of the protocone are 3.99, 5.68 and 7.95 µm, respectively. On the occlusal aspect of the protocone, they are slightly less (3.55, 4.94, 6.62 µm). Equivalent values in the paracone for inner, middle and outer thirds of occlusal enamel of equal thickness are slightly less than those for the protocone. The overall mean value of all cross striation spacings measured in the protocone is 5.56 µm (SD: 1.63 µm) and of all cross striation spacings in the paracone is 4.89 µm (SD: 1.24 µm). The overall mean value of cross striation spacings measured in the fastest trajectory of the protocone (palatal aspect) is 6.02 µm (SD: 1.75 µm) and in the fastest trajectory of the paracone (occlusal aspect) is 4.97 µm (SD: 1.51 µm).

Enamel formation rates at the EDJ. Measurements of daily enamel cross striation spacings in SK 835 were made in the same way at different positions along the EDJ between the dentine horn and the enamel cervix. At four positions spaced equally between the dentine horn and enamel cervix, measurements were made within the first 200 µm from the EDJ. The grand mean of 54 repeated 5-day averages within enamel 200 µm from the EDJ was 3.28 µm/day (SD: 0.310, range: 2.6-3.9 µm/day). This was rounded to 3.3 µm/day, which equals 61 days to form a 200-µm thickness of enamel close to the EDJ.

Total enamel formation time. It was not possible to image regular long-period striae of Retzius through the whole of the lateral enamel in either the protocone or the paracone. Moreover, counts of daily cross striations between regular long-period striae associated with perikymata at the enamel surface were inconsistent between the protocone and paracone. Direct counts between adjacent long-period striae in the protocone numbered 6 or 7 days (Supplementary Fig. S11). The average of five long-period stria widths was 31.9 µm and the average of ten 5-day groups of cross striations within these striae was 4.91 µm,

giving an average periodicity of 6.496 (6.5) days. It was not then possible to say if the periodicity is 6 or 7 days, since periodicities are by definition always expressed in whole days. Regions of the paracone cervix complicate this picture further. Here again, there are clear daily cross striations as well as alternating varicosities and constrictions with apparently well-expressed regular long-period increments, some of which can be associated with what might be construed as surface perikymata (Supplementary Fig. S11). However, the periodicity is twice, if not more than twice, that observed in the protocone cervix. Accordingly, in this study stria counts and long-period stria periodicity were not used in any calculations of crown formation time.

The methods used here for calculating total enamel formation time have been described in previous studies[16–19] and are derived from those originally described by Boyde[20,21], Risnes[22] and Antoine and co-workers[23]. These were used here to calculate cusp-specific enamel formation times for both the protocone and the incompletely preserved paracone. A 200-µm long prism path representing 61 days enamel formation was first measured from the tip of the dentine horn. From this point, a line was extended back to intersect with the EDJ either along or parallel with an accentuated incremental marking. The length of enamel along the EDJ from the dentine horn to this point of intersection formed in 61 days. This length along the EDJ, divided by 61 days, defines the extension rate between the dentine horn and the point of intersection at the EDJ. Again, from this point of intersection at the EDJ a second 200-µm prism path was extended out into enamel, and the same procedure repeated. Successive 61-day units of time defined in this way between the dentine horn and the enamel cervix were then cumulated to give a total cuspal enamel formation time. In the protocone there were 15 × 61 day 200-µm increments from cusp to cervix, plus a 30-day half increment which equals 15 × 61 + 30 = 945 days, or 2.59 years, of enamel formation. In the paracone, there were 9 × 61 day 200-µm increments = 549 days, or 1.50 years, of enamel formation to the incomplete enamel cervix.

Enamel extension rates. The extension rate is a measure of the rate at which new ameloblasts differentiated along the EDJ[24]. In the protocone, initial cuspal extension rates were 30 µm/day, dropping to 14 µm/day in the first 61 days of crown formation, and then to 7 µm/day by ~240 days. Thereafter, extension rates in this cusp remained constant at ~7 µm/day for the remainder of crown formation. In the paracone, extension rates were initially less at 21 µm/day, dropping immediately to 9 µm/day within 61 days, and then 6-7 µm/day as in the protocone. This slower extension rate in the paracone cusp tip than in the protocone may be an indication of slight section obliquity here clipping off the peak of the paracone dentine horn. This has implications for estimating paracone initiation time relative to the protocone.

Initiation of the paracone and protocone. The SXRF overview scans reveal a series of clear Sr bands both in the enamel and dentine of this specimen (Fig. 5). Alternating regions of Sr enrichment and depletion can be identified with more intense Sr bands superimposed upon this underlying pattern. Within the enamel and the root dentine, Sr bands allow cusps and roots forming at the same time to be cross-matched. In the crown, many of the Sr bands were associated with accentuated markings visible with transmitted light microscopy (TLM). Because the accentuated lines in enamel were more clearly defined and less diffuse than the Sr bands, they were used to align protocone and paracone formation. A number of accentuated lines, each of about equal intensity, run through the enamel. Most of these lines can be tracked back to the EDJ and matched to a time corresponding to the closest 61 day increment as defined above (Supplementary Fig. S7, white arrows). Almost all the accentuated lines appear bright in polarised light except a few strong lines that are dark. Lines that form within the cuspal enamel run between cusps,

but those that form after ~600 days are contained only within the lateral enamel and emerge at the protocone or paracone surface (Supplementary Fig. S7). In general, the mid-cuspal enamel and lateral enamel contain the most accentuated markings. Enamel formed between ~400 days and ~500 days and between ~670 days and ~850 days appeared worst affected by these, what are assumed to be, stress-related markings.

Four accentuated markings within enamel were identified in SK 835 that could be clearly seen running through the protocone, occlusal enamel and paracone (Supplementary Fig. S7, white arrows). These were used as common temporal registration markings. Daily cross striation counts from the EDJ to the first marking, and then between subsequent accentuated markings, were used to cross match enamel growth in the protocone with that in the paracone. Direct counts of daily incremental markings in the protocone to the first-formed bright accentuated line lying closest to the EDJ (Supplementary Fig. S7) were 210 days. Direct counts to the same corresponding line in the paracone were 190 days. This suggests the protocone began to mineralise ~20 days before the paracone. A second matching pair of accentuated lines occurs at 287 days in the protocone and 260 days in the paracone, suggesting a greater 27-day difference. It is unusual, but not unknown, for the protocone to initiate before the paracone and, on the face of it, that is what appears to have been the case in SK 835. But the difference observed of 20-30 days between the two cusps in this section is relatively small, and the error of making direct counts in decussating cuspal enamel, and with the possibility of some section obliquity at the paracone dentine horn, inclines to the safer assumption that both cusps initiated very close together in time. It follows that in the plots of EDJ length against tooth formation time both cusps were assumed to initiate at the same time (Figs 4, 7).

Supplementary Text S4: Dentine histology

Regions of interest. Digital photo-montages were first constructed of each root at low power between the most cervical and apically preserved root dentine of SK 835. Eight regions of interest were chosen that expressed good daily dentine increments between the root surface and to a depth of at least 200 µm into dentine (Supplementary Fig. S7, yellow boxes). These were in the mid-root and cervical third of the palatal root, the buccal and lingual mid-root, and the buccal apical region of the mesiobuccal root, and the mid-root and apical third of the distobuccal root. Higher power digital photomontages were constructed of these regions of interest.

Daily rates of root dentine formation. Daily dentine rates were measured in the same way as those in enamel. Six successive daily dentine increments were marked and the distance across them divided by 5. The mean of 340 such 5-day averages measured in 7 of the 8 regions of interest all within a 200-µm zone from the cement dentine junction (CDJ) was 2.49 µm (SD: 0.499, range: 1.62-4.0 µm). The average time to form a 200-µm thickness of root dentine from the CDJ was, therefore, estimated to be 200 / 2.49 = 80.32 days, which was rounded to 80 days (Supplementary Fig. S12). Additional measurements of daily dentine rates were possible between the CDJ and the lateral margin of the apical canal of the mesiobuccal root (Supplementary Fig. S7). Here, across 1040 µm of apical dentine, rates rose gradually away from the CDJ, and then reduced again towards the apical canal. The average rate was 2.87 µm/day (SD: 0.469, range: 1.78-3.65 µm/day). On this basis, apical closure from a stage of root formation equivalent to radiographic Root length complete (RC) could be estimated in this root.

Accentuated lines in the root dentine. A prominent Sr line visible in the SXRF scans was chosen as a marker of root formation common to all roots (Fig. 5 and Supplementary Fig. S7, orange arrows). A separate group of closely spaced accentuated lines visible in TLM can also be identified in the apical third of all three roots in TLM (Supplementary

Fig. S7, blue arrows). At higher power, these appear alternately either bright or dark in TLM, and are either more closely spaced or more widely separated from each other as if irregular periods of slowing are followed by catch-up periods of dentine formation (Supplementary Fig. S13). Together, they form a distinct pattern that can be accurately cross-matched between all three roots. In practice, the prominent Sr line visible in the SXRF images proved the most practical way to match root formation occurring at the same time.

Root formation in the palatal root from the protocone cervix (the last formed enamel) was calculated up to the Sr marker line. Root formation both before and beyond the Sr marker line, could also be calculated in the distobuccal root. The cervical enamel in the distobuccal root is poorly preserved and cannot be reliably linked to enamel in the rest of the crown. Moreover, the distobuccal root apex is incomplete, such that dentine in this root still falls short of the last formed apical root dentine (Fig. 5 and Supplementary Fig. S7). However, the distobuccal root contains the longest record of root growth and can be aligned with the other two forming roots using the Sr marker line. Only the mesiobuccal root has a clear well-formed closed apex, but this root does not preserve an enamel cervix and is a slightly shorter root. Root formation in the mesiobuccal root can, however, also be cross-matched with root formation in the other two roots using the Sr marker band. Distobuccal root formation from the accentuated Sr marker line (Fig. 5 and Supplementary Fig. S7, orange arrows) to the radiographic stage of Root length complete (RC) was estimated using 80-day time segments along the CDJ (see below). However, the final period of dentine formation (Apex closure) was estimated from direct counts of daily increments across the thickness of the apical primary dentine in the mesiobuccal root. In this way, an estimate of total root formation time from the protocone enamel cervix to the accentuated Sr marker line (Supplementary Fig. S7, orange arrows), and then onwards in the distobuccal root and finally through mesiobuccal root to apical closure, could be represented as a composite plot of increasing tooth height along the EDJ against tooth formation time (Fig. 4).

Total root formation time. In the palatal root, from the junction of the last formed enamel and first formed root dentine at the cervix, a line was drawn along the direction of dentine tubules 200 μm deep into dentine. From this point, accentuated growth lines in dentine, marking the former position of the forming dentine front, were used to track a second line back to intersect the CDJ at the root surface. The length along the CDJ, between the start point at the cervix and this intersection at the CDJ, took 80 days to form. Starting again from this point of intersection at the CDJ, the process was repeated as far as possible towards the root apex. The sum of the consecutive 80-day time segments equals the total root formation time when both the enamel cervix and the apical root margin are preserved. This procedure is illustrated in Supplementary Fig. S14 and was repeated along the outer CDJ in each of the three roots of SK 835, but was only possible within the limitations of root preservation.

There were 9 consecutive 80-day segments in the palatal protocone root to the fractured root end (9 × 80 = 720 days, or 1.97 years). However, beyond protocone enamel completion at 945 days, there were only 6 × 80-day segments prior to the Sr marker line (7 × 80 = 560 days, or 1.53 years). The Sr line in the palatal and distobuccal roots can therefore be cross-matched at 945 + 560 = 1505 days, or 4.123 years (Supplementary Fig. S7, orange arrows). In the distobuccal root, there were a total of 19 × 80-day segments from a reconstructed but poorly defined enamel cervix to the fractured root apex (19 × 80 = 1520 days, or 4.16 years). However, there were only 12 × 80-day segments (12 × 80 = 960 days, or 2.63 years) beyond the Sr line in the distobuccal root, excluding apex closure. Total tooth formation time prior to apex closure in the distobuccal root then equals 1505 + 960 = 2465 days, or 6.75 years. If average rates of root dentine formation were

slightly lower or higher (75 or 85 days to form 200 µm rather than 80 days) root completion would have been 6.75 years ± 95 days. It was not possible to estimate apex closure time in the distobuccal root. However, in the mesiobuccal root, dentine thickness between the CDJ and the lateral aspect of the completed apical canal measured 1040 µm. At an average daily formation rate of 2.87 µm/day measured within this region, this is equivalent to 362 days, or about a year. What this might have been in the likely slightly longer and usually last-completed palatal root remains unknown. It may also have taken less time than this for the distobuccal root apex to close completely. Nonetheless, it suggests that total tooth formation time would almost certainly have been greater than 7 years, and even as much as 7.75 years in SK 835.

Figure 4 shows the cumulative growth curve for increasing distance along the EDJ/CDJ (µm) against formation time (years) expressed here as a 5th order polynomial that allows formation time (Y) to be predicted from a known value of EDJ/CDJ length (X) as follows:

$$Y = .018 + 8.875e^{-5}X + 4.414e^{-8}X^2 - 3.009e^{-12}X^3 + 4.581e^{-17}X^4 + 6.494e^{-22}X^5. \quad \text{(Eq. 2)}$$
$$(R^2 = .993)$$

Supplementary Fig. S15 represents the incremental growth of SK 835 expressed differently, with successive ages given from the time of M3 initiation shown for ¼ fractions of time for crown and root formation, and ¼ fractions of height for crown and root formation.

Root extension rates. As in enamel, the root extension rate (in this case, the rate at which new odontoblasts differentiate along the CDJ) can be estimated by dividing a defined length of the CDJ by the time taken to form it. The length of the CDJ formed in each 80-day segment varies considerably in SK 835. Close to the cervix, root dentine extension rate calculations in all three roots were close to the rates calculated at the end of enamel formation (~9 µm/day). Thereafter there was a swift rise to a peak rate of 15 µm/day, in the mesiobuccal root, and to 19 µm/day, in both the palatal and distobuccal roots between 3.5 and 4 years after protocone initiation. Following this, rates slowed again towards the root apex. The last of the 80-day segments of the mesiobuccal root, just prior to apex formation, extended at 3-5 µm/day. Thereafter, the strongly curving contour of the rounded root apex, combined with poor expression of accentuated forming-front markings, makes extension rate estimates beyond the stage of root length complete (RC) less reliable by this method. The time taken for apical closure of the mesiobuccal and distobuccal roots was therefore estimated on the basis of direct counts of daily dentine increments and an observed average daily rate in apical dentine of 2.87 µm/day.

Supplementary Text S5: Enamel thickness assessment.
By using µXCT records, the 2D relative enamel thickness (RET) and absolute crown strength (ACS) indices were comparatively assessed in SK 835 and in other *P. robustus* and extant hominid M3s (Supplementary Table S3). 2D RET provides an estimation of crown enamel proportions[25], while ACS gives an estimation of tooth strength, i.e., of resistance to fracture[26]. The biplot of 2D RET vs. ACS is shown in Supplementary Fig. S16. The extant apes have thin to moderately thin enamel (as attested by their 2D RET values), associated with low ACS values. Modern humans, located below the regression line, also have low ACS values, suggesting that their thick enamel (comparable to the 2D RET values of *P. robustus*) are less adapted to resist fractures than the thick enamelled condition of *P. robustus* (including SK 835), the latter taxon exhibiting markedly higher ACS values.

Supplementary Tables

Supplementary Table S1. Radiographic scores for lower teeth represented in Supplementary Figs S1-S3.

Taxon	Number	Pm3	Pm4	M1	M2	M3
Gorilla	18911	Ri	R1/4	RC	Ri	Crypt small
Gorilla	1939-937	CC	Ri	A1/2	CC	Ci
Gorilla	29112	CC	CC	RC	CC	Ci
Gorilla	Os Hill RCS	C3/4	CC	R3/4	CC	Ci
Gorilla	1268/650	C3/4	C3/4	R3/4	C3/4	Crypt small
Pongo	1948-831	CC	CC	RC	CC	Ci
Pongo	1852-3212	CC	CC	RC	CC	Ci
Pongo	1976-1410	Ri	R1/4	A1/2	Ri	Ci
Pongo	A65-13	CC	CC	R3/4	CC	Crypt large
Pan	M675	R1/4	R1/4	A1/2	Ri	Ci
Pan	1939-1002	CC	CC	A1/2	CC	Ci
Pan	M855	CC	Ri	RC	CC	Crypt large
Pan	M507	R1/4	R1/4	A1/2	Ri	Ci
Pan	M847	Ri	Ri	A1/2	Ri	Ci

Supplementary Table S2. Predicted formation times for successive cumulative 1 mm lengths along the EDJ and CDJ in SK 835 M3.

EDJ/CDJ length (µm)	Predicted time (years)	Predicted time (days)
1000	0.044	16
2000	0.310	113
3000	0.602	220
4000	0.914	334
5000	1.239	452
6000	1.570	573
7000	1.900	693
8000	2.223	811
9000	2.532	924
10000	2.825	1031
11000	3.095	1130
12000	3.343	1220
13000	3.565	1301
14000	3.764	1374
15000	3.943	1439
16000	4.106	1499
17000	4.260	1555
18000	4.417	1612

EDJ/CDJ length (µm)	Predicted time (years)	Predicted time (days)
19000	4.588	1675
20000	4.790	1748
21000	5.042	1840
23000	5.790	2113
24000	6.343	2315
25000	7.060	2577
26000	7.979	2912

Supplementary Table S3. Relative enamel thickness (2D RET) and absolute crown strength (ACS) indices assessed in *P. robustus* and extant hominid M3s.

Taxon	Specimen	2D RET	ACS	References
P. robustus	SK 13-14	36.53	4.54	original data
P. robustus	SK 31	22.71	3.91	original data
P. robustus	SK 48	29.64	4.32	original data
P. robustus	SK 49	29.57	4.23	original data
P. robustus	SK 105	25.32	3.94	original data
P. robustus	SK 831a	22.36	3.81	original data
P. robustus	SK 835	28.95	3.99	original data
P. robustus	SK 836	24.27	4.17	original data
P. robustus	SKW 11	25.96	4.27	original data
P. robustus	TM 1517C	30.57	4.12	original data
H. sapiens	ind1	25.77	2.50	original data
H. sapiens	ind2	28.69	2.80	original data
H. sapiens	ind3	26.17	2.94	original data
H. sapiens	ind4	23.17	2.33	original data
Pan	IPS5698	11.36	1.62	original data
Pan	MCZ-9493	13.62	1.60	[27]
Pan	MCZ-23167	13.87	1.98	[27]
Pan	MCZ-38018	12.06	1.74	[27]
Gorilla	MCZ-14750	14.01	2.80	[27]
Gorilla	MCZ-37264	12.81	2.48	[27]
Gorilla	MCZ-37266	15.88	2.43	[27]
Gorilla	MCZ-38326	10.65	2.40	[27]
Pongo	Sbg_903	14.16	2.24	original data
Pongo	MZS-2632	13.78	2.47	original data
Pongo	MNSNT Pongo1	14.29	2.25	original data
Pongo	IPS9031	16.86	2.42	original data

Supplementary Table S4. Plio-Pleistocene hominins in which M3 development is incomplete ordered here by M3 stage that best approximates the proportion of crown or root height/length formed. Stages were estimated from the literature and/or from casts/images. A minimum and a maximum relative dental age (RDA in years) based on M3 initiation at 4 years and at 7 years respectively, likely bracket the true age at death.

Specimen accession number	Taxon	Provenance	M3 stage	Min M3 RDA	Max M3 RDA
StW 151[11]	*A. africanus*	South Africa	Crypt	4.0	7.0
KNM-ER 820[14,28,29]	early *Homo*	Kenya	Crypt	4.0	7.0
Sts 8[30]	*A. africanus*	South Africa	CC	2.59+4=6.59	2.59+7=9.59
MH1[31–33]	*A. sediba*	South Africa	CC	2.59+4=6.59	2.59+7=9.59
SK 843[30]	*P. robustus*	South Africa	CC	2.59+4=6.59	2.59+7=9.59
KNM-WT 17400[34]	*P. boisei*	Kenya	CC	2.59+4=6.59	2.59+7=9.59
KNM-ER 62000[35]	*H. rudolfensis*	Kenya	CC	2.59+4=6.59	2.59+7=9.59
KNM-WT 15000[36]	*H. erectus* s.l.	Kenya	CC	2.59+4=6.59	2.59+7=9.59
A.L. 486-1[37]	*A. afarensis*	Ethiopia	R1/2	4.26+4=8.26	4.26+7=11.26
TM 1511[30]	*A. africanus*	South Africa	R1/2	4.26+4=8.26	4.26+7=11.26
SK 13/14[30]	*P. robustus*	South Africa	R1/2	4.26+4=8.26	4.26+7=11.26
DNH 10[38]	*P. robustus*	South Africa	R1/2	4.26+4=8.26	4.26+7=11.26
OH 13[30,39]	*H. habilis*	Tanzania	R1/2	4.26+4=8.26	4.26+7=11.26
OH 16[39]	*H. habilis*	Tanzania	R1/2	4.26+4=8.26	4.26+7=11.26
D2700 / D2735[40,41]	*H. erectus* s.l.	Georgia	R1/2	4.26+4=8.26	4.26+7=11.26
Sts 52/53[30]	*A. africanus*	South Africa	R3/4	5.04+4=9.09	5.04+7=12.04
TM 1517[42]	*P. robustus*	South Africa	R3/4	5.04+4=9.04	5.04+7=12.04
OH 5[30,43]	*P. boisei*	Tanzania	R3/4	5.04+4=9.04	5.04+7=12.04
KNM-CH1[44]	*P. boisei*	Kenya	R3/4	5.04+4=9.04	5.04+7=12.04
KNM-ER 992[28,29]	*H. erectus* s.l.	Kenya	A1/2	6.67+4=10.67	6.67+7=13.67
Sangiran 4[45,46]	*H. erectus* s.l.	Indonesia	A1/2	6.67+4=10.67	6.67+7=13.67

Supplementary Figures

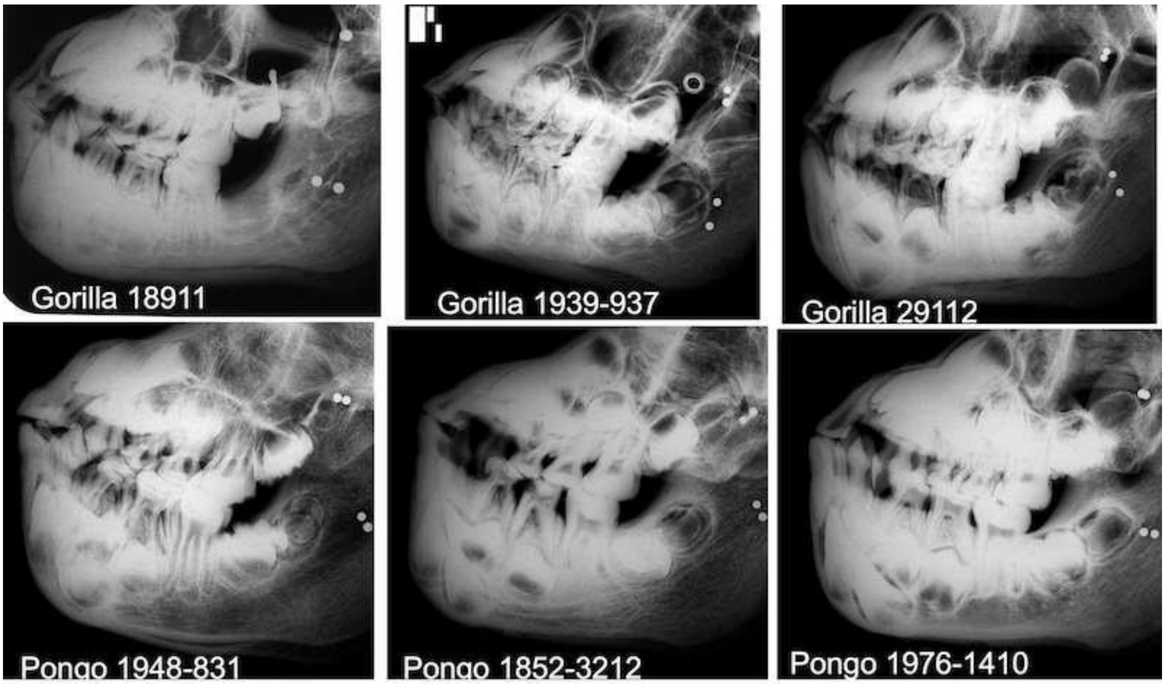

Supplementary Figure S1. *Gorilla* and *Pongo* at the stage of upper and/or lower M3 initiation[4].

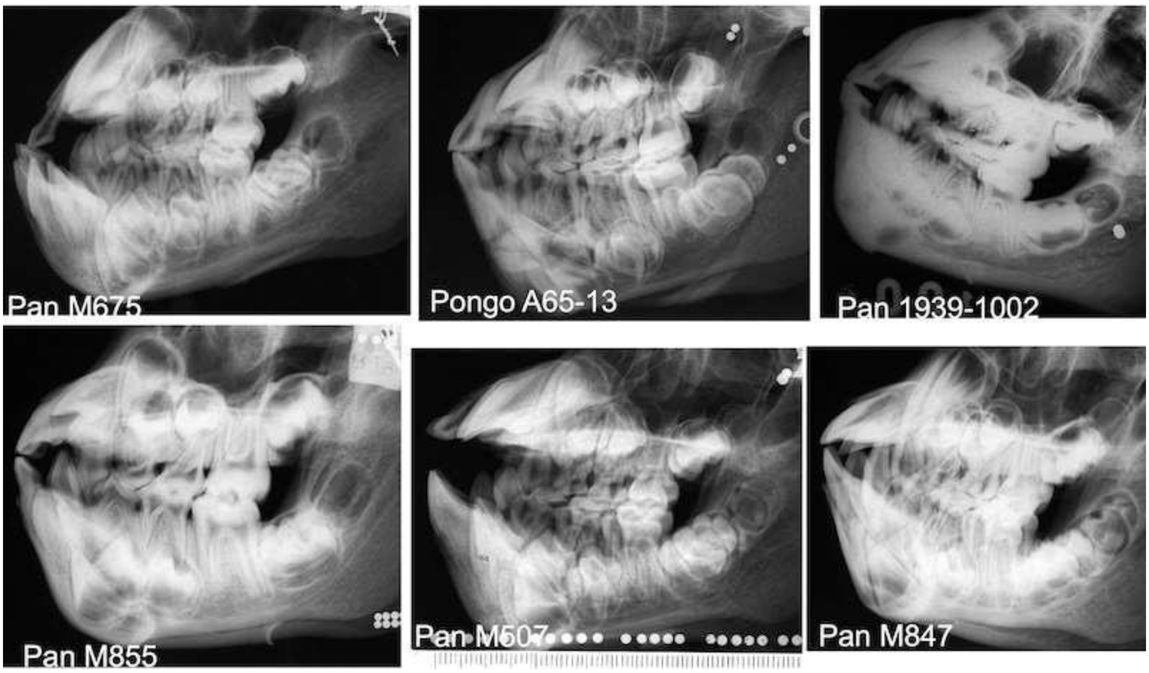

Supplementary Figure S2. *Pan* and *Pongo* at the stage of upper and/or lower M3 initiation[4].

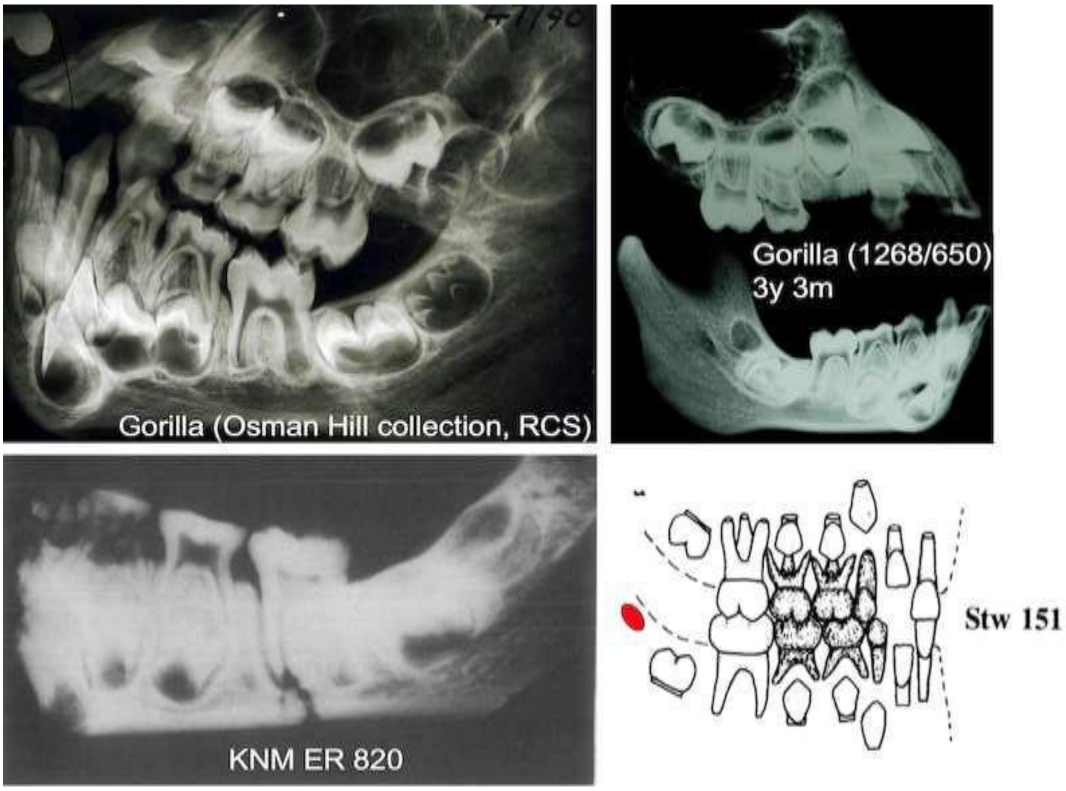

Supplementary Figure S3. Two *Gorilla* specimens as well as early *Homo* KNM-ER 820[14,29] and *A. africanus* drawing of specimen StW 151 (adapted from [11]) at stages close to M3 initiation.

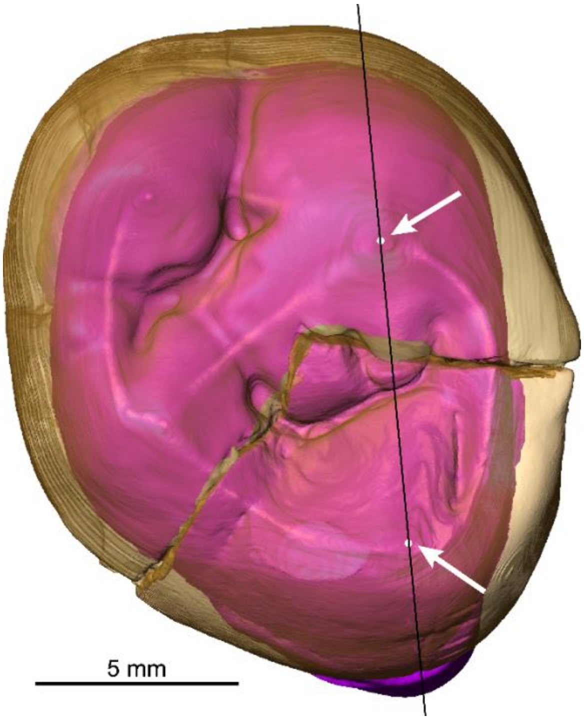

Supplementary Figure S4. µXCT-based 3D image of SK 835 with both the mesial dentine horns (arrows) in the plane of the histological section (line).

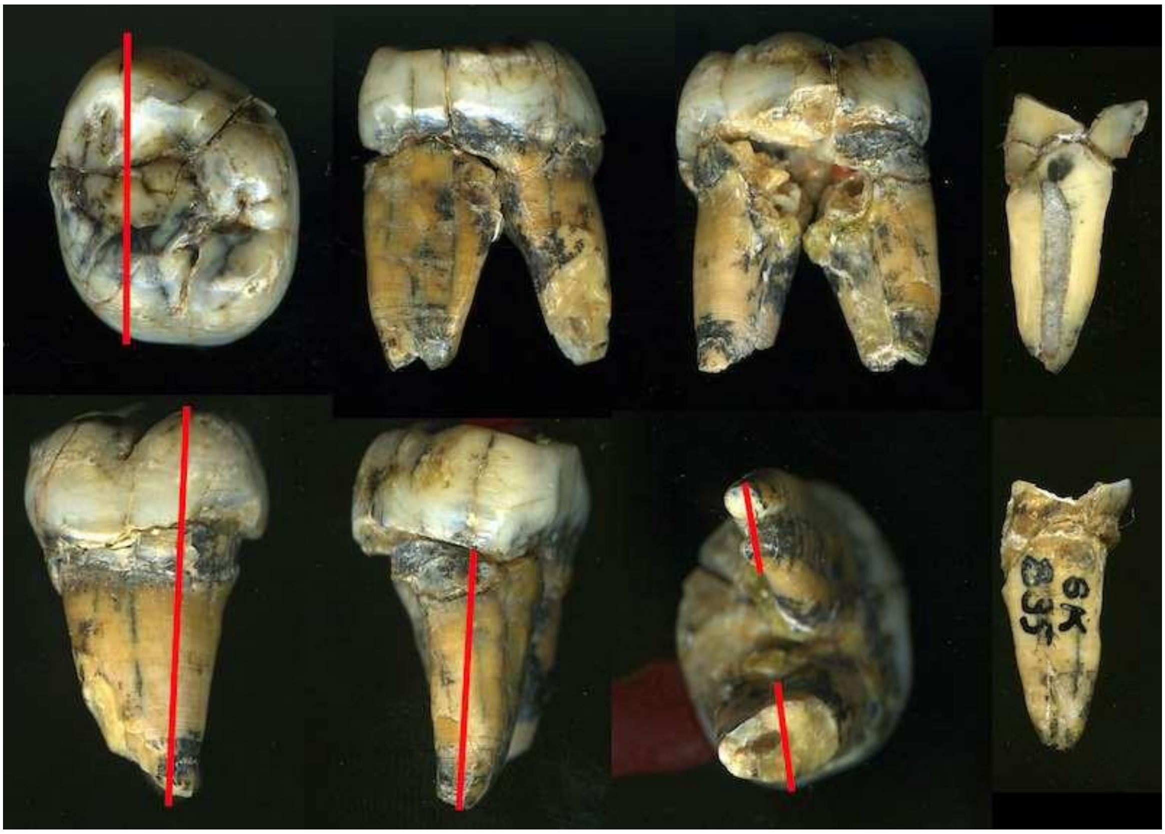

Supplementary Figure S5. SK 835: planes of section (in red) through the crown and roots.

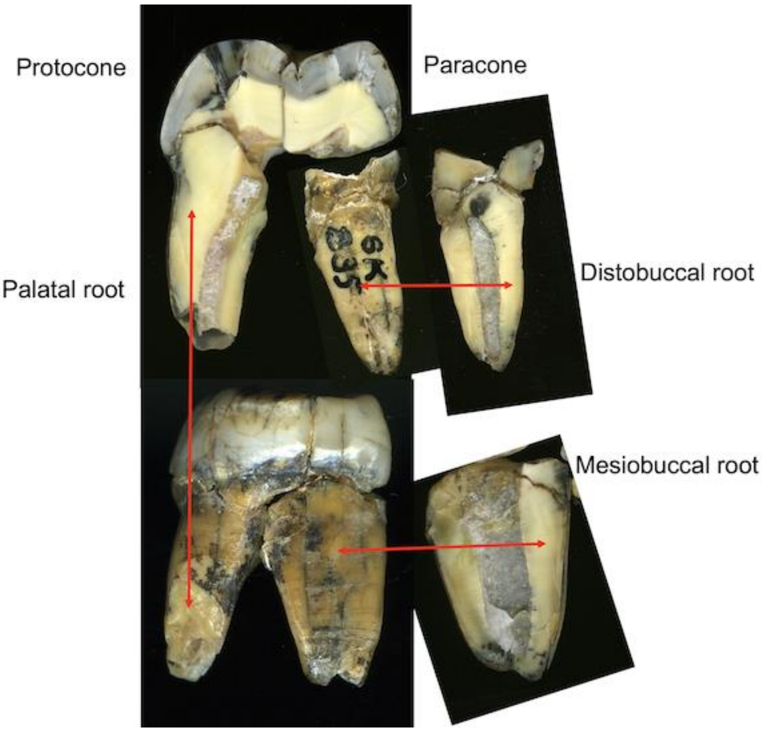

Supplementary Figure S6. SK 835: the polished block faces of the crown and roots aligned together.

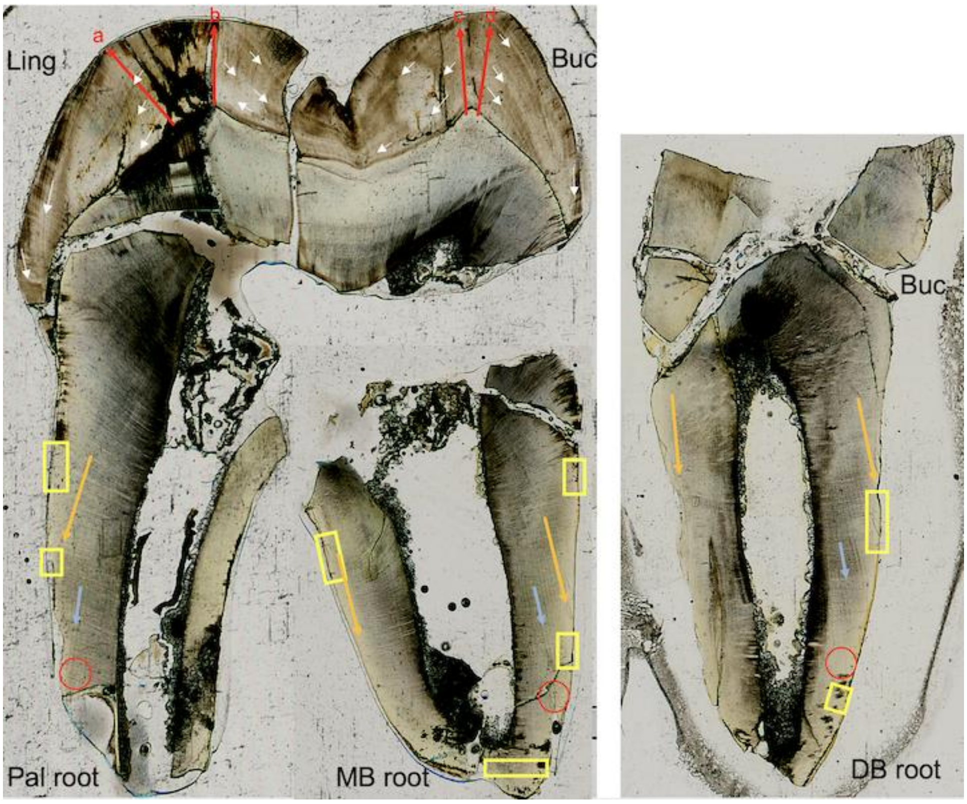

Supplementary Figure S7. SK 835: three ground sections aligned together. Mesial section showing the palatal root to the very left beneath the protocone, and the mesio-buccal root to the right positioned beneath the paracone on the buccal aspect of the tooth. The disto-buccal root appears separately to the far right (See Supplementary Fig. S6). Matching accentuated lines in enamel (white arrows). Occlusal enamel trajectories ('a'-'d' red arrows). Regions where daily dentine rates were identified and measured (yellow boxes). Position of prominent strontium lines in roots (orange arrows; see Sr map in Fig. 5). Matching accentuated lines in roots (blue arrows and red circles and at higher power in these regions in Supplementary Fig. S13). Buc: buccal; DB root: distobuccal root; Ling: lingual; MB root: mesiobuccal root; Pal root: palatal root.

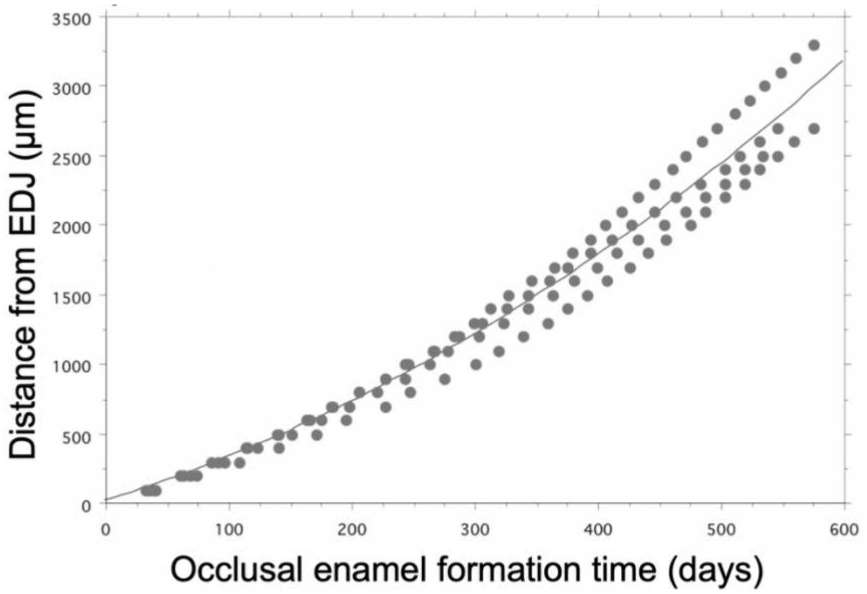

Supplementary Figure S8. SK 835: four occlusal trajectories (indicated by red arrows in Supplementary Fig. S7) with best fit line. Eq. 1: $Y = 12.651 + .284X - 3.459e^{-5}X^2$ where Y is time in days.

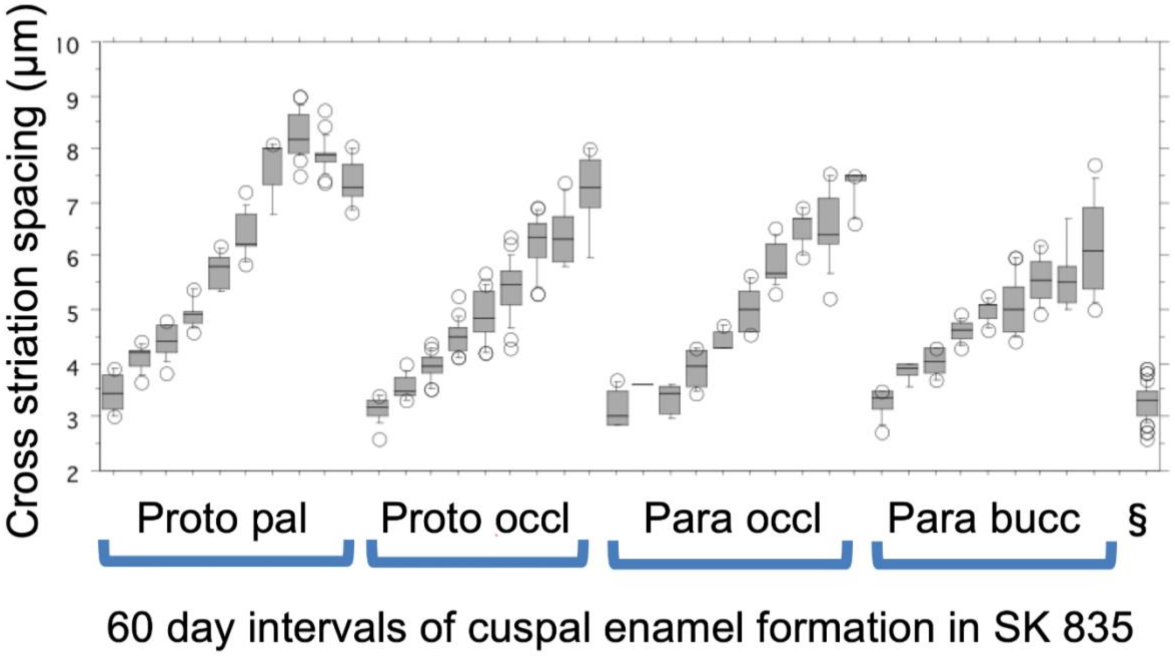

Supplementary Figure S9. SK 835: gradients of increasing enamel formation rates at successive 60-day intervals along four occlusal tracks in the protocone and paracone as indicated in Supplementary Fig. S7 (red arrows ‘a’, ‘b’, ‘c’, ‘d’). ‘§’ represents all measurements made within 200 µm of the EDJ. The EDJ is to the left and the outer enamel surface to the right in all four cuspal plots.

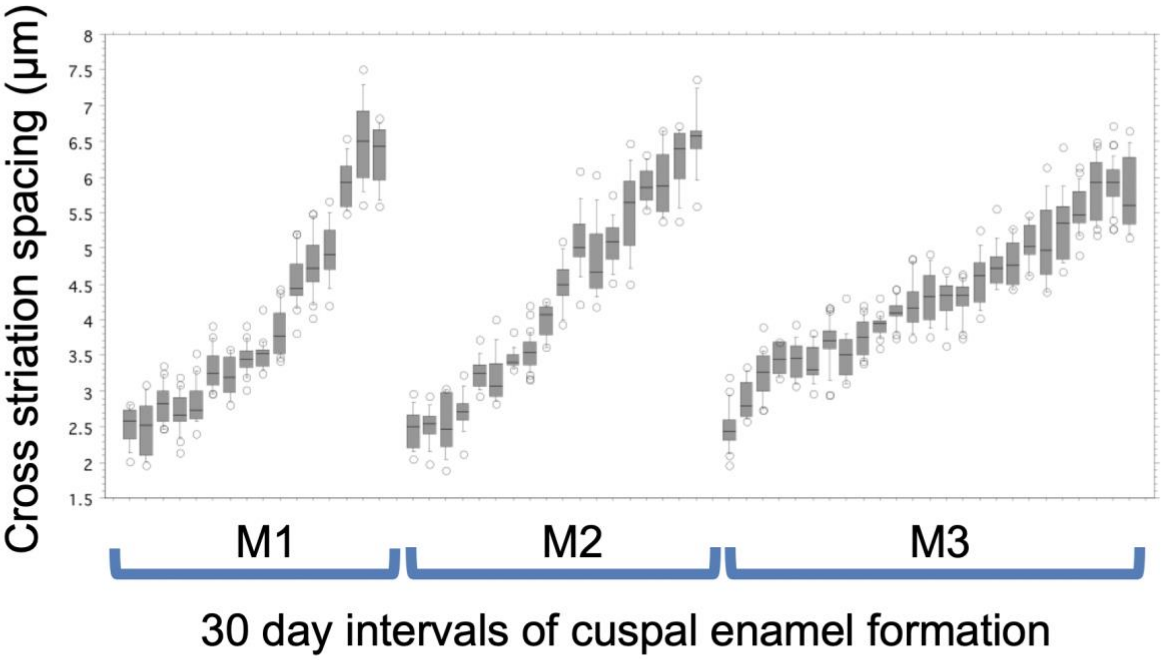

Supplementary Figure S10. Gradients of increasing occlusal enamel formation rates in three modern human molars from the same individual (30-day intervals). The EDJ is to the left and the outer enamel surface to the right in all three cuspal plots.

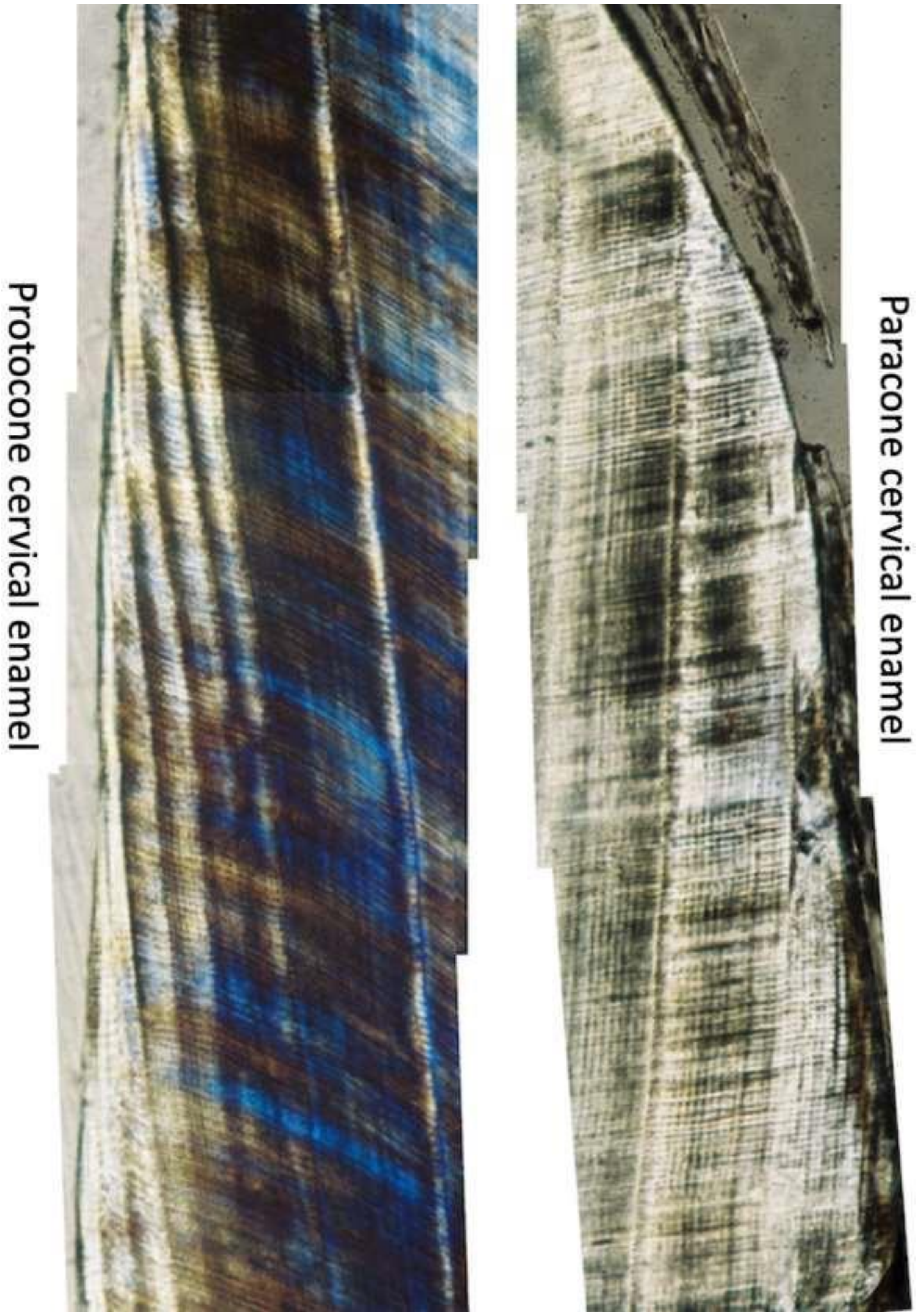

Supplementary Figure S11. SK 835: daily cross striations and long-period incremental markings towards the cervical aspect of the lateral enamel (buccal paracone and palatal protocone). Apparently inconsistent periodicities, as well as indistinct regions where they were difficult to identify and count, excluded their use for any estimates of enamel formation times.

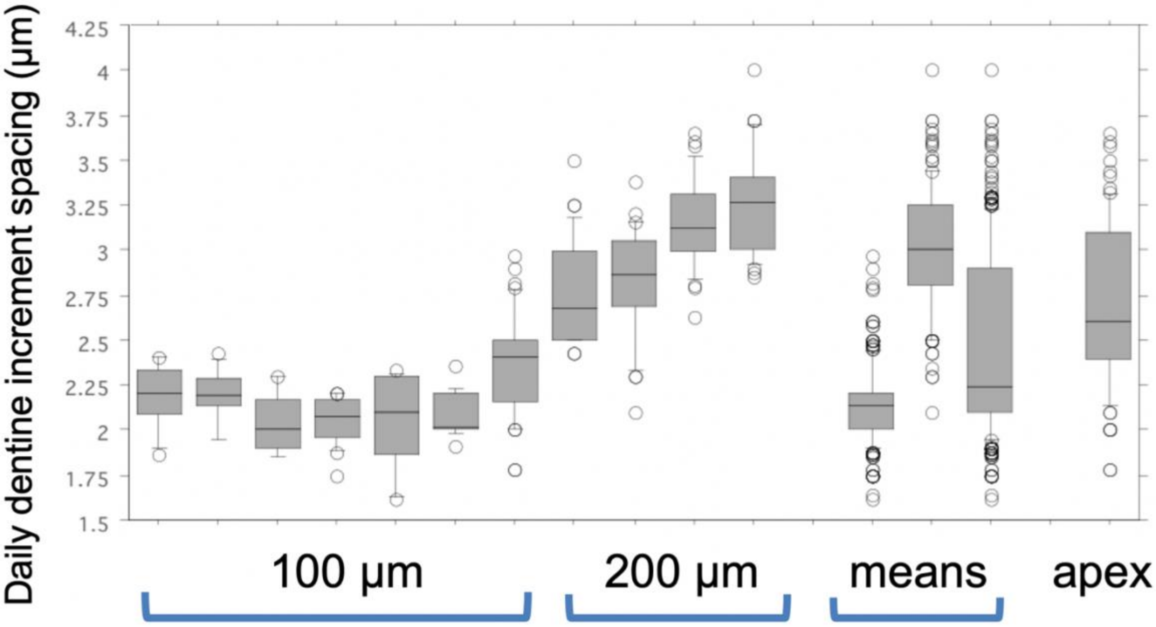

Supplementary Figure S12. SK 835: daily root dentine increment spacing (µm) close to the CDJ with means for 100 µm, 200 µm and both zones averaged, as well as spacings measured in the apical root dentine (see Supplementary Fig. S7 for key).

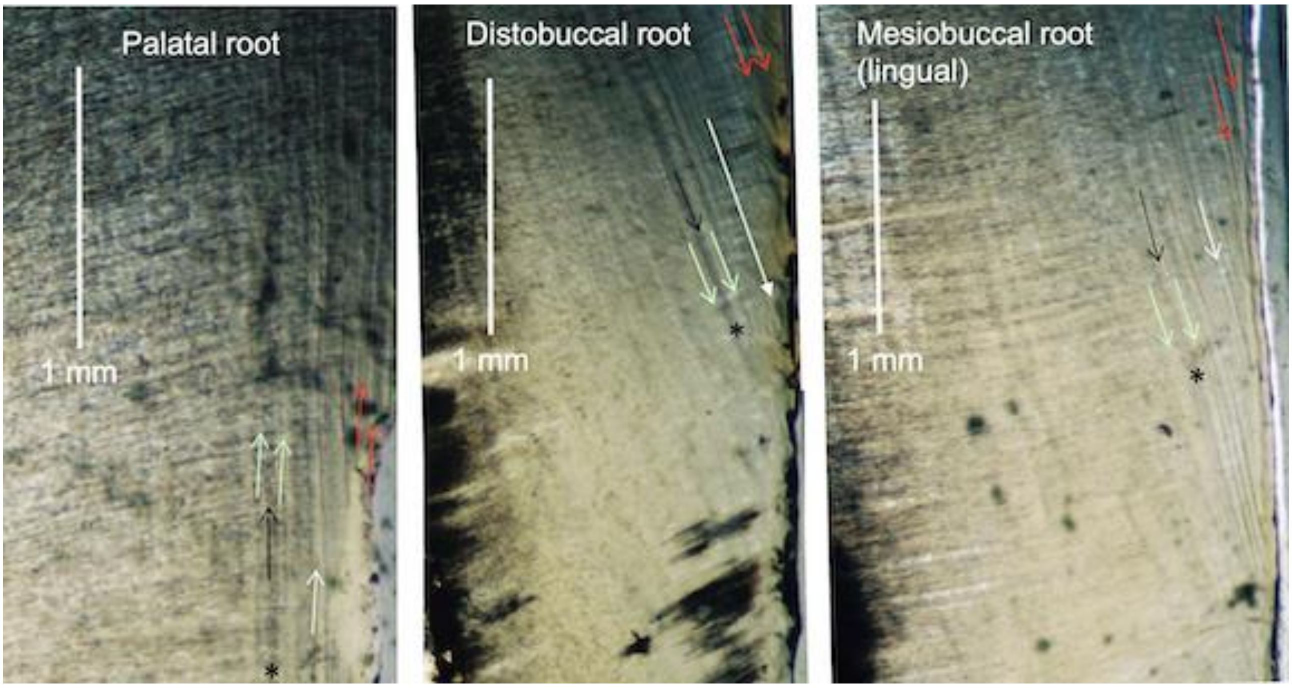

Supplementary Figure S13. SK 835: transmitted light micrographs of matching patterns of accentuated markings in root dentine. The red circles in Supplementary Fig. S7 indicate the locations of these examples.

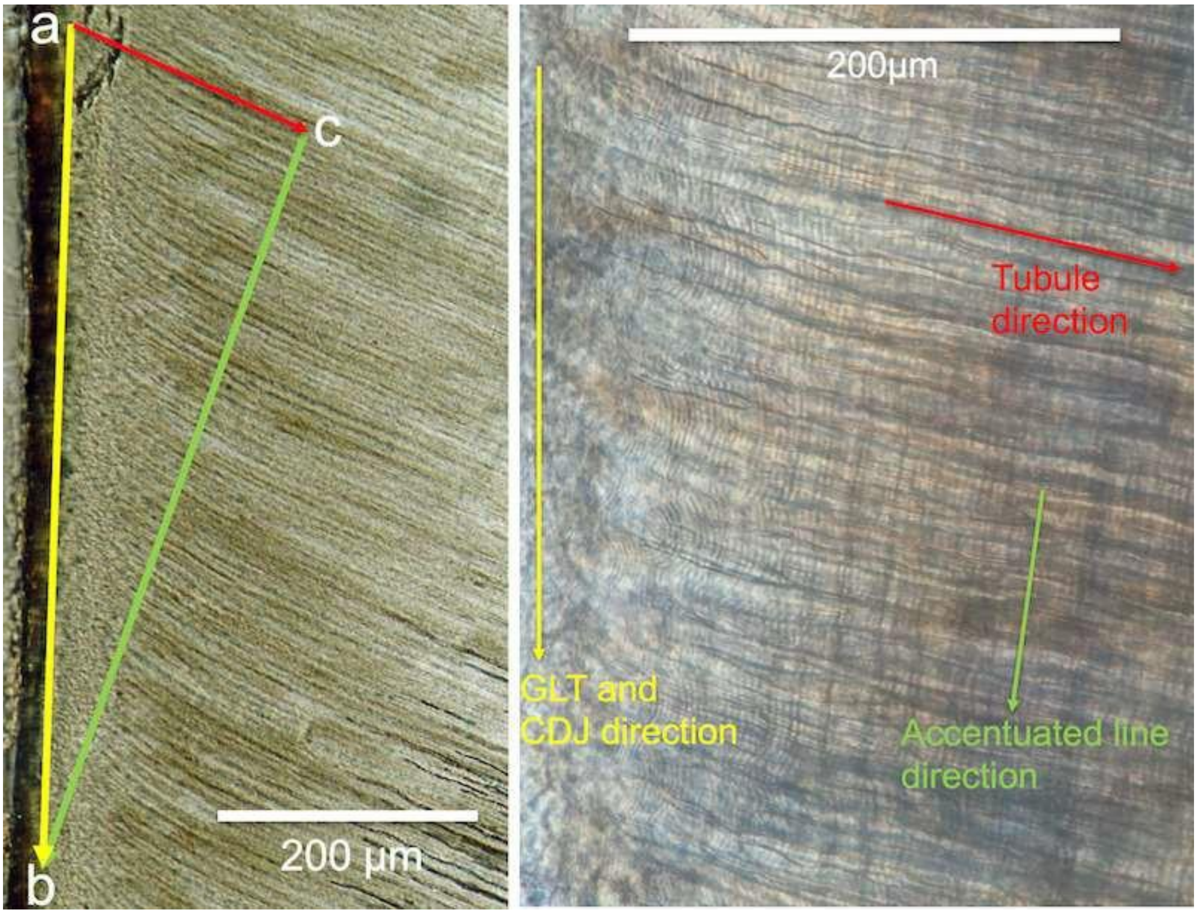

Supplementary Figure S14. SK 835. (**left**) The yellow arrow in root dentine runs just deep to cementum along the granular layer of Tomes (GLT) at the cementum dentine junction (CDJ), yellow arrow in both images, towards the root apex. The red arrow in both images runs in the direction of dentine tubules and the green arrow in both images runs in the direction of accentuated markings. The red line a-c is 200 µm long. The time taken to form a-c (200 µm; average: 80 daily increments) also equals the time taken to extend between a-b (extension rate). (**right**) The annotated higher power image also shows each of these features but alongside daily root dentine increments and accentuated markings at higher resolution.

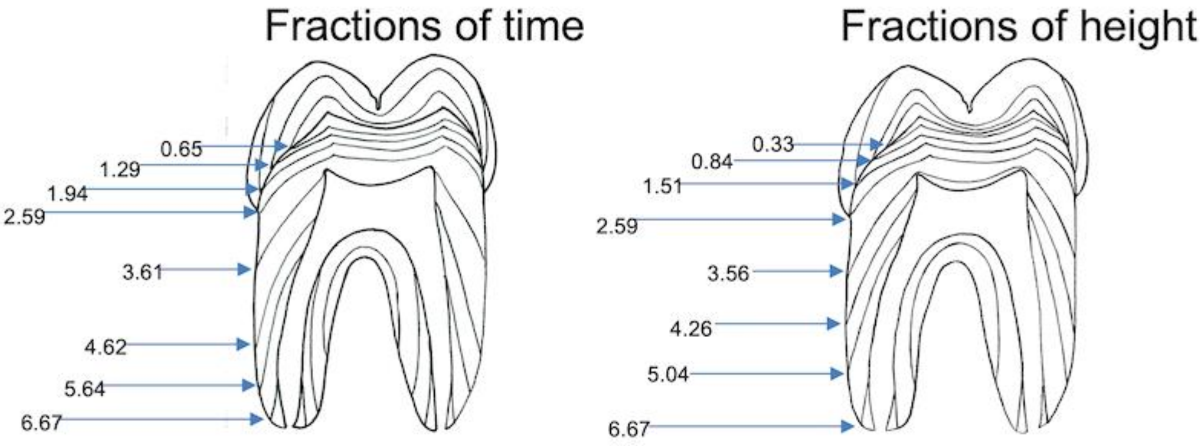

Supplementary Figure S15. Reconstructed M3 based on SK 835 showing incremental markings at ¼ fractions of crown and root formation time and ¼ fractions of crown and root height. Times of attainment (years) are arrowed along the EDJ and then along the CDJ.

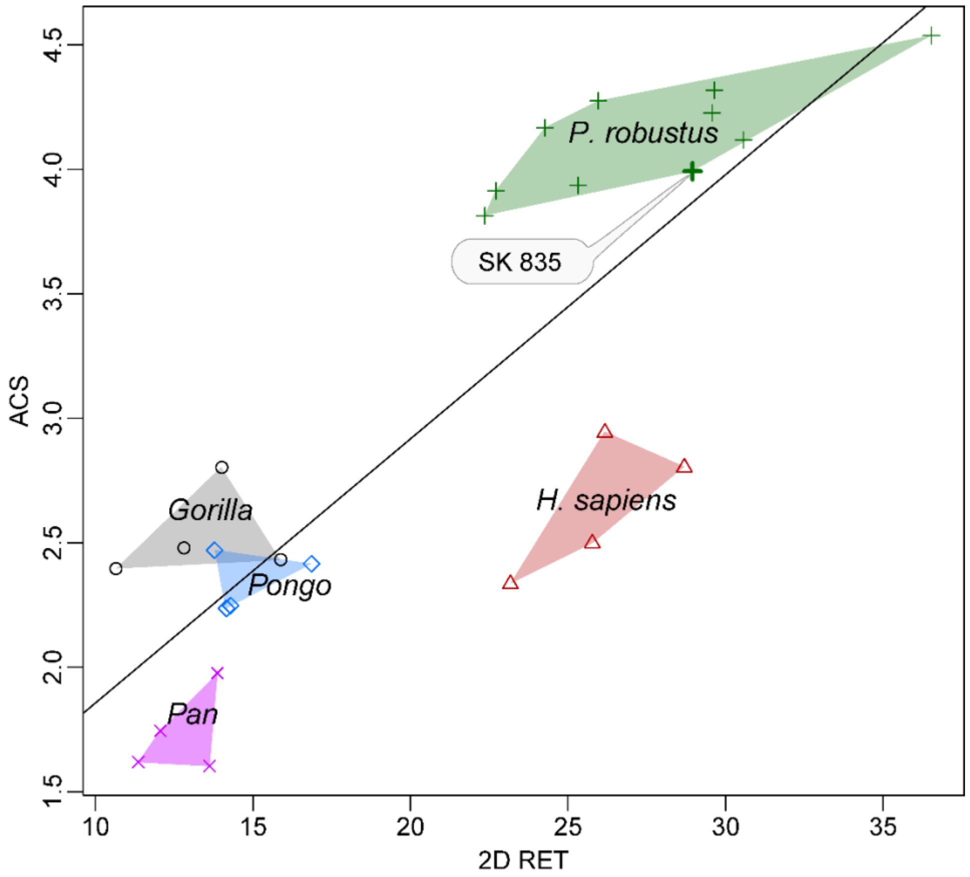

Supplementary Figure S16. Bivariate plot of ACS against 2D RET in *P. robustus* (including SK 835) and extant hominid M3s with the regression line computed between the values for the two indices (Y = 0.795 + 0.106X where X is 2D RET; R^2=0.67).

Supplementary References

1. Liversidge, H. M. Timing of human mandibular third molar formation. *Ann. Hum. Biol.* **35**, 294–321 (2008).
2. Liversidge, H. M. Predicting mandibular third molar agenesis from second molar formation. *Acta Stomatol. Croat.* **42**, 311–317 (2008).
3. Beynon, A. D., Dean, M. C. & Reid, D. J. Histological study on the chronology of the developing dentition in gorilla and orangutan. *Am. J. Phys. Anthropol.* **86**, 189–203 (1991).
4. Dean, C. & Wood, B. A digital radiographic atlas of great apes skull and dentition. in *Digital Archives of Human Paleobiology, ADS Solutions* vol. 3 (L. Bondioli, R. Macchiarelli, Editors, 2003).
5. Schwartz, G. T., Reid, D. J., Dean, M. C. & Zihlman, A. L. A Faithful Record of Stressful Life Events Recorded in the Dental Developmental Record of a Juvenile Gorilla. *Int. J. Primatol.* **27**, 1201–1219 (2006).
6. Kuykendall, K. L. Dental development in chimpanzees (*Pan troglodytes*): The timing of tooth calcification stages. *Am. J. Phys. Anthropol.* **99**, 135–157 (1996).
7. Anemone, R. L., Mooney, M. P. & Siegel, M. I. Longitudinal study of dental development in chimpanzees of known chronological age: Implications for understanding the age at death of Plio-Pleistocene hominids. *Am. J. Phys. Anthropol.* **99**, 119–133 (1996).
8. Kralick, A. E. *et al.* A radiographic study of permanent molar development in wild Virunga mountain gorillas of known chronological age from Rwanda. *Am. J. Phys. Anthropol.* **163**, 129–147 (2017).
9. Reid, D. J. & Guatelli-Steinberg, D. Updating histological data on crown initiation and crown completion ages in southern Africans. *Am. J. Phys. Anthropol.* **162**, 817–829 (2017).
10. Dean, M. C., Beynon, A. D., Thackeray, J. F. & Macho, G. A. Histological reconstruction of dental development and age at death of a juvenile *Paranthropus robustus* specimen, SK 63, from Swartkrans, South Africa. *Am. J. Phys. Anthropol.* **91**, 401–419 (1993).
11. Moggi-Cecchi, J., Tobias, P. V. & Beynon, A. D. The mixed dentition and associated skull fragments of a juvenile fossil hominid from Sterkfontein, South Africa. *Am. J. Phys. Anthropol.* **106**, 425–465 (1998).
12. Le Cabec, A., Tang, N. K. & Tafforeau, P. Accessing developmental information of fossil hominin teeth using new synchrotron microtomography-based visualization techniques of dental surfaces and interfaces. *PLoS One* **10**, e0123019 (2015).
13. Smith, T. M. *et al.* Dental Ontogeny in Pliocene and Early Pleistocene Hominins. *PLoS One* **10**, e0118118 (2015).
14. Dean, M. C. The dental developmental status of six East African juvenile fossil hominids. *J. Hum. Evol.* **16**, 197–213 (1987).
15. Beynon, A. D. & Dean, M. C. Distinct dental development patterns in early fossil hominids. *Nature* **335**, 509–514 (1988).
16. Dean, M. C. A histological method that can be used to estimate the time taken to form the crown of a permanent tooth. in *Forensic Microscopy for Skeletal Tissues: Methods and Protocols* (ed. Bell, L. S.) vol. 915 89–100 (Humana Press, Springer Science and Business Media, 2012).

17. Guatelli-Steinberg, D., Floyd, B. A., Dean, M. C. & Reid, D. J. Enamel extension rate patterns in modern human teeth: Two approaches designed to establish an integrated comparative context for fossil primates. *J. Hum. Evol.* **63**, 475–486 (2012).
18. Dean, M. C. & Cole, T. J. Human Life History Evolution Explains Dissociation between the Timing of Tooth Eruption and Peak Rates of Root Growth. *PLoS One* **8**, e54534 (2013).
19. Dean, M. C., Humphrey, L., Groom, A. & Hassett, B. Variation in the timing of enamel formation in modern human deciduous canines. *Arch. Oral Biol.* **114**, 104719 (2020).
20. Boyde, A. Estimation of age at death of young human skeletal remains from incremental lines in the dental enamel. *Excerpta Medica Int. Congr. Ser.* **80**, 36–46 (1963).
21. Boyde, A. Developmental interpretations of dental microstructure. *Primate Life Hist. Evol. Monogr. Primatol.* **14**, 229–267 (1990).
22. Risnes, S. Enamel apposition rate and the prism periodicity in human teeth. *Eur. J. Oral Sci.* **94**, 394–404 (1986).
23. Antoine, D., Hillson, S. & Dean, M. C. The developmental clock of dental enamel: a test for the periodicity of prism cross-striations in modern humans and an evaluation of the most likely sources of error in histological studies of this kind. *J. Anat.* **214**, 45–55 (2009).
24. Shellis, R. P. Variations in growth of the enamel crown in human teeth and a possible relationship between growth and enamel structure. *Arch. Oral Biol.* **29**, 697–705 (1984).
25. Martin, L. Significance of enamel thickness in hominoid evolution. *Nature* **314**, 260–263 (1985).
26. Schwartz, G. T., McGrosky, A. & Strait, D. S. Fracture mechanics, enamel thickness and the evolution of molar form in hominins. *Biol. Lett.* **16**, 20190671 (2020).
27. *Morphosource Database* http://www.morphosource.org (2020).
28. Leakey, R. E. F. & Wood, B. A. New evidence of the genus *Homo* from East Rudolf, Kenya. II. *Am. J. Phys. Anthropol.* **39**, 355–368 (1973).
29. Wood, B. A. *Koobi Fora research project. Clarendon*. vol. Volume 4: Hominid cranial remains. (Clarendon Press, 1991).
30. Skinner, M. F. & Sperber, G. H. *Atlas of Radiographs of Early Man*. (Alan R. Liss, 1982).
31. Berger, L. R. *et al. Australopithecus sediba*: A new species of *Homo*-like australopith from South Africa. *Science* **328**, 195–204 (2010).
32. Le Cabec, A., Tafforeau, P., Smith, T. M., Carlson, K. J. & Berger, L. R. Dental Development of the *Australopithecus sediba* Juvenile MH1 Determined from Synchrotron Virtual Paleohistology. in *Proceedings of the European Society for the study of Human Evolution 3* 103 (2014).
33. ESRF. *Australopithecus Sediba at the ESRF*. (2010).
34. Leakey, R. E. F. & Walker, A. New *Australopithecus boisei* specimens from east and west Lake Turkana, Kenya. *Am. J. Phys. Anthropol.* **76**, 1–24 (1988).
35. Leakey, M. G. *et al.* New fossils from Koobi Fora in northern Kenya confirm taxonomic diversity in early *Homo*. *Nature* **488**, 201–204 (2012).
36. Brown, F., Harris, J., Leakey, R. & Walker, A. Early *Homo erectus* skeleton from west Lake Turkana, Kenya. *Nature* **316**, 788–792 (1985).
37. Kimbel, W. H. & Delezene, L. K. “Lucy” redux: A review of research on *Australopithecus*

afarensis. *Am. J. Phys. Anthropol.* **140**, 2–48 (2009).

38. Moggi-Cecchi, J., Menter, C., Boccone, S. & Keyser, A. Early hominin dental remains from the Plio-Pleistocene site of Drimolen, South Africa. *J. Hum. Evol.* **58**, 374–405 (2010).
39. Tobias, P. *Olduvai Gorge, volume 4: The skulls, endocasts and teeth of* Homo habilis. (Cambridge University Press, 1991).
40. Lordkipanidze, D. *et al.* Postcranial evidence from early *Homo* from Dmanisi, Georgia. *Nature* **449**, 305–310 (2007).
41. Vekua, A. *et al.* A New Skull of Early *Homo* from Dmanisi, Georgia. *Science* **297**, 85 (2002).
42. Cazenave, M. *et al.* Reassessment of the TM 1517 odonto-postcranial assemblage from Kromdraai B, South Africa, and the maturational pattern of *Paranthropus robustus*. *Am. J. Phys. Anthropol.* **172**, 714–722 (2020).
43. Tobias, P. V. *The cranium and maxillary dentition of* Australopithecus (Zinjanthropus) boisei. vol. Olduvai Gorge. Vol. II (Cambridge University Press, 1967).
44. Carney, J., Hill, A., Miller, J. A. & Walker, A. Late Australopithecine from Baringo District, Kenya. *Nature* **230**, 509–514 (1971).
45. Schwartz, J. H. & Tattersall, I. *The Human Fossil Record. Craniodental Morphology of Genus* Homo *(Africa and Asia)*. vol. 2 (John Wiley & Sons, 2003).
46. Zanolli, C. *et al.* Evidence for increased hominid diversity in the Early-Middle Pleistocene of Java, Indonesia. *Nat. Ecol. Evol.* (2019) doi:10.1038/s41559-019-0860-z.
